# Hydrogel-based tumor embolization and synergistic therapeutic strategies

**DOI:** 10.1016/j.bioactmat.2025.12.031

**Published:** 2025-12-20

**Authors:** Yisheng Peng, Xiuyi Wu, Hui Liu, Fengyi Yang, Xu Cheng, Mengmeng Miao, Shangqing Chen, Kaifei Yan, Hui Zheng, Hongwei Cheng, Gang Liu

**Affiliations:** a State Key Laboratory of Vaccine for Infectious Diseases, Xiang An Biomedicine Laboratory, National Innovation Platform for Industry-Education Integration in Vaccine Research, State Key Laboratory of Molecular Vaccinology and Molecular Diagnostics, School of Public Health, Xiamen University, Xiamen, 361102, China; b Department of Interventional Radiology, Fujian Medical University Union Hospital, Fuzhou, 350001, China; c College of Materials Science and Engineering, Huaqiao University, Xiamen, 361021, China

**Keywords:** Hydrogel embolization, Transarterial embolization, Drug delivery, Combination therapy, Clinical translation

## Abstract

Hydrogels, characterized by their porous network structures and microenvironment-responsive properties, have been widely explored for tumor embolization. Physically or dynamically crosslinked hydrogels-such as ionic, hydrogen-bonded, or supramolecular systems-exhibit favorable microcatheter injectability and shear-thinning behavior, whereas covalently crosslinked systems are typically delivered as low-viscosity precursors for in situ gelation. These features endow embolic hydrogels with tunable drug delivery capacity and excellent biocompatibility, enabling precise occlusion of tumor-feeding arteries and controlled, localized therapeutic release. This review uniquely emphasizes the innovative design of multifunctional hydrogels, focusing on their role in synergistic multimodal therapies and personalized cancer treatment. It provides a comprehensive overview of the latest advancements in the preparation methods and functional properties of embolic hydrogels, alongside their emerging clinical applications. Additionally, we address the challenges hindering clinical translation and propose future directions, including the personalized design of intelligent hydrogels and the exploration of synergistic mechanisms for multimodal therapeutic strategies. This review offers valuable insights into the design, development, and clinical application of embolic hydrogels for precision medicine.

## Highlights

• This review highlights innovative design strategies in embolizing hydrogels for enhanced tumor therapy.
• It explores hydrogels' multifunctionality, combining embolization with various therapies to enhance treatment outcomes.
• The review discusses the current challenges and future prospects of embolizing hydrogels in personalized cancer treatments.

## Graphical abstract

**Figure:**
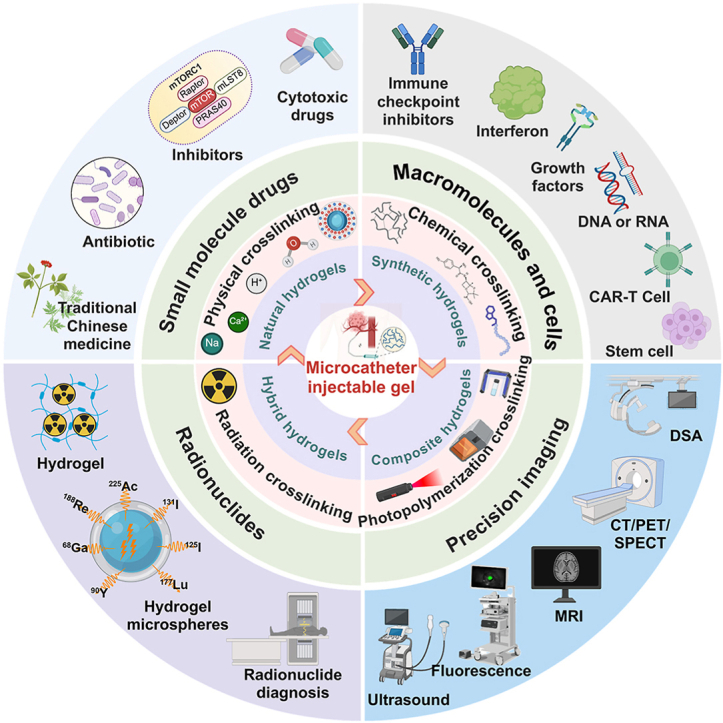

## 1 Introduction

With the continuous advancement of minimally invasive interventional technologies, tumor treatment has increasingly shifted from traditional open surgery to minimally invasive embolization therapy, typically performed under imaging guidance [1,2]. This therapy involves delivering embolic agents through an intravascular route to block the blood supply to the tumor. In the treatment of hepatocellular carcinoma (HCC), locally injected embolic agents effectively obstruct the tumor's blood flow, thereby slowing tumor growth [[3], [4], [5]]. Embolic materials used in tumor interventional therapy are generally categorized into solid and liquid embolic agents [6,7]. Iodized oil, the most widely used liquid embolic agent, is preferred for its excellent fluidity and favorable digital subtraction angiography (DSA) imaging properties [8,9]. However, iodized oil has poor stability and mechanical properties, and it is easily washed away by blood, limiting its efficacy in tumor treatment [10,11]. In contrast, solid embolic materials, such as glass microspheres and coils, are favored for their superior embolic properties [12,13]. Despite these advantages, solid embolic materials have limited plasticity, making it difficult to effectively embolize small blood vessels and posing a risk of ectopic embolism [[14], [15], [16]].

Embolic hydrogels are materials that typically contain more than 90 % water, with a viscoelastic network structure and reversible gelation properties. In some applications, embolic hydrogels can also take the form of microspheres, allowing for injection through microcatheters. These embolic hydrogels, with their unique physical and chemical properties, are used as embolic agents in tumor treatment. The three-dimensional network structure of hydrogels provides them with excellent embolic performance and drug loading capacity [17,18]. Additionally, embolic hydrogels exhibit good biocompatibility and degradability, making them promising for tumor interventional therapy [19,20]. By incorporating imaging agents such as iodized oil, tantalum nanoparticles, ultrasound imaging nanoparticles, and magnetic nanoparticles into hydrogels, researchers have developed visual hydrogel materials with imaging capabilities. These materials enable precise tumor embolization under imaging guidance using techniques such as DSA, Computed Tomography (CT), Magnetic Resonance Imaging (MRI), and ultrasound [[21], [22], [23]]. Moreover, the three-dimensional network structure of hydrogels serves as a platform for the delivery of small molecules, macromolecular drugs, functionalized cells, radionuclides, and immunotherapies, facilitating the combined use of multiple treatment modalities to enhance therapeutic outcomes [[24], [25], [26]].

Despite the numerous advantages of embolic hydrogels, their clinical application still faces significant challenges, including issues related to stability, injectability, catheter blockage, ectopic embolization, biocompatibility, and large-scale commercial production. This review emphasizes the innovative role of hydrogels in tumor embolization therapy, particularly their ability to integrate with synergistic therapeutic strategies to enhance treatment outcomes. Unlike traditional embolic materials, hydrogels offer unique advantages due to their highly adaptable properties, including a three-dimensional network structure, tunable viscosity, and the ability to incorporate therapeutic agents for controlled release.

**Fig. 1:**
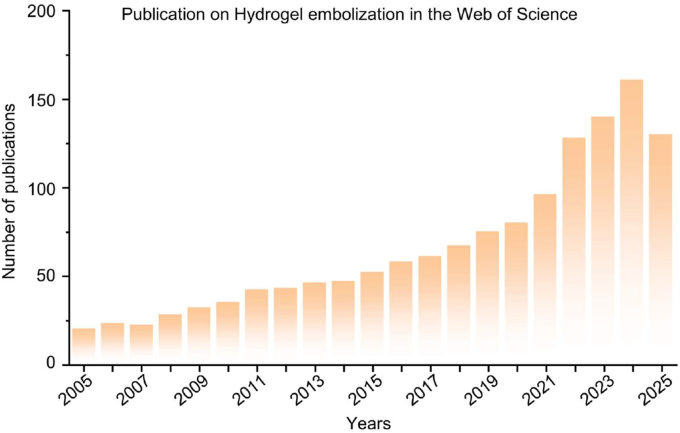
Annual publication output on hydrogel-based embolization research (2005–2025). The number of publications per year was retrieved from the Web of Science Core Collection (SCI-Expanded and SSCI) using the following keywords: (“hydrogel” AND “embolization”), (“hydrogel microspheres” AND “embolization”), (“hydrogel” AND “tumor embolization”), (“hydrogel embolic agent”), and (“hydrogel-based embolization therapy”). Timespan was limited to 2005–2025, and document types included Articles and Reviews. The upward trend indicates growing research interest in hydrogel-based embolic materials and synergistic therapeutic strategies.

The increasing interest in hydrogel-based tumor embolization is reflected in the growing number of publications on this topic over the past two decades. As shown in Fig. 1, the number of publications on hydrogel embolization has steadily increased since 2005, with a notable surge in recent years, particularly after 2020. This trend highlights the expanding research and development in this field, underscoring the increasing importance and potential of hydrogel-based materials in tumor embolization and interventional therapy.

**Fig. 2:**
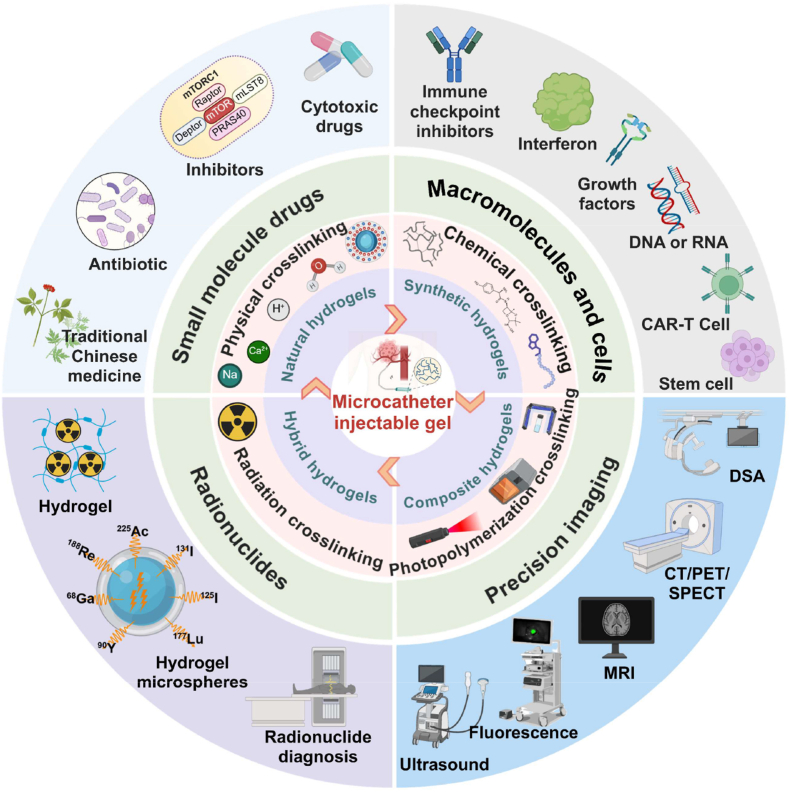
Schematic representation of hydrogels for tumor embolization and drug delivery. The diagram illustrates the synthesis of hydrogels and their diverse applications in drug delivery. Created with BioRender.com.

The review systematically explores recent advancements in hydrogel-based tumor embolization, providing an overview of various hydrogel preparation methods (Fig. 2). It further investigates their applications in tumor embolization therapy across different tumor types and discusses their potential in synergistic treatments such as chemotherapy, radiotherapy, hyperthermia, imaging, robotic precision embolization, tumor microenvironment (TME) regulation, and immunotherapy. Additionally, this article identifies the technical challenges faced by embolic hydrogels and evaluates the prospects for clinical translation, emphasizing their promise in personalized and multimodal therapy strategies. The review highlights future directions for research, including the personalized design of intelligent hydrogels and the development of synergistic mechanisms in multimodal therapies, to further optimize tumor treatment strategies and accelerate their clinical application.

## 2 Synthesis method of embolic hydrogel

The preparation methods of embolic hydrogels are pivotal in determining their structural frameworks and functional performance, directly influencing their effectiveness in tumor embolization therapy. While the crosslinking mechanism fundamentally defines the formation of hydrogel networks, the physicochemical properties-including injectability, mechanical strength, and bio-adhesion-play equally crucial roles in ensuring safe and efficient transcatheter delivery and long-term embolic stability. Therefore, understanding how synthetic strategies affect these properties is essential for the rational design of hydrogel embolic systems.

In clinical embolization, hydrogels are typically delivered through microcatheters with inner diameters of only 0.3–0.6 mm. This requires materials that exhibit excellent injectability, allowing smooth passage under shear without clogging or premature gelation [6,27]. Injectability is primarily determined by the rheological behavior of the hydrogel precursor, including viscosity, shear-thinning capacity, and sol-gel transition kinetics. Ideally, embolic hydrogels should maintain low viscosity under shear stress (during injection) and rapidly recover their elasticity once delivered to the target vessel, forming a stable occlusion in situ [28,29]. Shear-thinning or thermosensitive systems-such as Poly(N-isopropylacrylamide) (pNIPAAm)-based or physically crosslinked chitosan hydrogels-have demonstrated favorable injectability and controllable gelation under physiological conditions, enabling precise delivery and reducing the risk of catheter blockage.

Beyond injectability, mechanical properties critically influence the durability and safety of vascular occlusion. The embolic hydrogel must possess sufficient elasticity and compressive strength to withstand hemodynamic shear forces, intraluminal pressure, and pulsatile blood flow, while remaining compliant enough to adapt to the vessel geometry. Insufficient mechanical stability may lead to embolus fragmentation, migration, or recanalization, whereas excessive stiffness may damage vascular endothelium. Typically, embolic hydrogels are designed with compressive moduli in the range of 10–100 kPa, which matches the compliance of soft vascular tissues [30,31]. Mechanical strength can be tailored by adjusting polymer concentration, crosslinking density, or by adopting dual crosslinking strategies (e.g., combining physical and chemical bonds) to reinforce the network without compromising injectability [30,32,33].

Bio-adhesive properties are also essential for ensuring the firm retention of the hydrogel at the embolization site, especially under high-flow conditions in tumor-feeding arteries. Hydrogels with intrinsic or engineered tissue adhesiveness can adhere to vascular endothelium or perivascular tumor tissues via hydrogen bonding, electrostatic interaction, or covalent coupling through catechol or phenolic moieties. For example, chitosan-based, dopamine-functionalized PEG, and tannic acid-modified hydrogels have demonstrated enhanced interfacial adhesion and prolonged retention at the embolization site [17,34,35]. Such adhesive interactions not only improve embolic stability but also help localize co-delivered therapeutic agents, enhancing treatment precision and durability. Consequently, when evaluating hydrogel synthesis strategies, it is critical to consider how each crosslinking mechanism influences these key physicochemical properties. In addition to conventional physical or chemical crosslinking methods, the sol-gel reaction has recently emerged as an alternative strategy for designing embolic materials. For instance, tantalum alkoxide-based precursors can undergo in situ sol-gel transition within the vasculature, forming a stable inorganic-organic hybrid network that achieves effective vessel occlusion and radiopacity simultaneously [36]. This approach provides a controllable and minimally invasive pathway for endovascular embolization, expanding the material design toolbox for hydrogel-based embolic systems.

To optimize the performance of hydrogel embolic materials in tumor embolization, it is essential to thoroughly understand how different crosslinking mechanisms influence their key physicochemical properties. Common crosslinking strategies used in the synthesis of embolic hydrogels include physical crosslinking, chemical crosslinking, radiation-induced crosslinking, and photopolymerization [37,38]. Each of these methods offers distinct advantages and limitations in terms of key parameters such as injectability, gelation control, mechanical stability, and bioadhesion. A detailed comparison of these crosslinking techniques, with regard to their impact on the functional performance and clinical applicability of embolic hydrogels, is summarized in Table 1. Additionally, recent advancements in hydrogel microsphere synthesis have opened new avenues for enhancing the embolic efficacy and precision of hydrogel-based therapies. The use of hydrogel microspheres, formed through techniques such as emulsion polymerization or coacervation, provides a promising approach for controlled drug delivery and targeted embolization. Microspheres offer several advantages, including tunable size, high surface area for drug loading, and the ability to localize therapeutic agents directly at the site of embolization, thus improving therapeutic outcomes. The development of hydrogel microspheres with tailored mechanical properties and enhanced bioadhesion can further improve their stability within the vasculature, while minimizing the risk of embolus migration or fragmentation.

**Table 1:** Advantages and disadvantages of various cross-linking methods for embolic hydrogels.

Type	Mechanism	Advantage	Disadvantage	Reference
Physical crosslinking	Hydrogen bonding	Simple preparation method (fast cross-linking time and mild preparation temperature) No cross-linking agent required Good biocompatibility Reversibility and tunability	Poor long-term stability and mechanical strength; easy to dissociate; difficult to precisely adjust crosslink density and pore size	[42,44,45]
	Hydrophobic interactions	Enhanced mechanical properties Self-healing ability Anti-swelling properties Durable embolization stability	Complex manufacturing process Temperature resistance limit Mechanical performance limitations Poor adjustability	[35,53,56]
	Ionic/electrostatic interactions	Improved elasticity Self-healing ability Anti-swelling properties Good durability and long-lasting performance	Dependence on environmental conditions Limited mechanical strength Immune response Sensitivity to specific ions	[[61], [62], [63], [64]]
Chemical crosslinking	Schiff base reactions	Rapid and efficient crosslinking Good biocompatibility Reversible and dynamically tunable network	Poor stability Poor fatigue resistance and brittleness resistance Strict reaction conditions (byproduct formation, reproducibility, and batch-to-batch variability) Potential toxicity issues	[28]
	Michael addition reactions	Mild and efficient reaction conditions Stable crosslinked network Biocompatible and dynamically reversible	Slow reaction rate Difficulty in controlling crosslinking degree and degradation rate Potential toxicity issues	[119]
	Click chemistry	Highly efficient and selective reaction mild, catalyst-assisted process Good biocompatibility	Limited selection of reactants Possible catalyst toxicity or residue Sensitivity to reaction conditions; high cost	[27,270]
Radiation crosslinking	–	Efficient cross-linking process No chemical reagents added, environmentally friendly Excellent adjustability and adaptability Biocompatibility	High cost of equipment Potential radiation damage Difficulty in controlling reaction uniformity	[[87], [88], [89],^92^]
Photopolymerization crosslinking	–	Spatially and temporally controllable process Mild and fast crosslinking No organic solvents or toxic catalysts Good biocompatibility	Limited light penetration depth Potential photosensitizer toxicity Difficulty in controlling degradation and uniformity	[73,[80], [81], [82]]

Overall, selecting the appropriate crosslinking strategy is crucial for optimizing the mechanical, rheological, and adhesive properties of hydrogel embolic systems, ensuring their effectiveness in tumor treatment. In the following sections, we will explore the specific details and advantages of the most commonly used crosslinking approaches, as well as recent advancements in hydrogel microsphere synthesis, which further contribute to the design of highly efficient and stable embolic materials.

### 2.1 Physical crosslinking

Physically crosslinked embolic hydrogels form a three-dimensional network through reversible, non-covalent interactions. These interactions primarily involve hydrogen bonding, hydrophobic interactions, ionic/electrostatic interactions, and host-guest recognition. Compared to chemical crosslinking systems, physically crosslinked hydrogels offer higher biomedical safety, as they do not require chemical crosslinking agents during preparation. This results in a reduced risk of toxicity due to chemical agent residues [39]. Moreover, the gelation process for this type of hydrogel typically occurs under mild conditions, without the need for strict temperature or pH control, and does not rely on complex synthesis steps. Consequently, this makes them particularly suitable for encapsulating and delivering bioactive substances such as drugs, proteins, and cells. This also supports in situ injection and *in vivo* gelation treatment strategies. Despite the significant advantages in biocompatibility and ease of preparation, physically crosslinked hydrogels have some limitations. Since their crosslinking mainly relies on reversible interactions, the resulting gels tend to have lower mechanical strength, structural stability, and resistance to dilution compared to chemical crosslinked hydrogels [40]. To overcome these limitations in practical applications, polymer structure design or enhanced synergistic crosslinking methods are often used to improve the physical properties of these hydrogels, making them better suited for the complex mechanical demands of interventional embolization therapy. These hydrogels exhibit shear-thinning behavior, allowing for easy microcatheter injection, while their temperature sensitivity enhances controlled *in vivo* gelation [11,41]. The gelation kinetics are critical in embolization therapy, enabling rapid formation of stable embolic plugs at target sites and optimizing embolization efficacy. Furthermore, their thermal responsiveness adapts to tumor or vascular microenvironments, enabling localized drug release and effective occlusion of tumor-feeding arteries.

Hydrogen bonding, an important form of non-covalent interaction, plays a critical role in the formation of hydrogels [42]. Through hydrogen bonding, polymer chains or polymers can form a reversible three-dimensional network structure with water molecules under mild conditions. This allows for in situ gelation and enhances the physical stability of hydrogels. Importantly, this mechanism does not require complex crosslinking conditions and can often be achieved at room temperature or in physiological environments. As a result, it is widely utilized in the design of injectable hydrogels. The number, distribution, and strength of hydrogen bonds significantly influence the physical and chemical properties of hydrogels, including mechanical strength, drug loading capacity, and swelling behavior. Among the various hydrogen bond-based hydrogel systems, pNIPAAm is one of the most extensively studied materials for hydrogen bond-based hydrogels. Its molecular structure features both hydrophilic amide groups (-CONH-) and hydrophobic isopropyl groups (-CH(CH_3_)_2_), which impart significant temperature sensitivity [43]. This makes pNIPAAm a versatile material for temperature-responsive hydrogels. The TempSLE hydrogel, primarily composed of pNIPAAm, forms a three-dimensional network copolymer of N-isopropylacrylamide and N-propylacrylamide [44]. At room temperature (25 °C), pNIPAAm remains in a hydrophilic liquid state. Upon exposure to human body temperature, the solution transitions to a hydrophobic phase, causing the polymer chains to collapse and form a gel-like structure [45]. This temperature-sensitive behavior is crucial for its application in drug delivery and embolization therapies. For pNIPAAm-based hydrogels, the gelation time typically ranges from several minutes to around 10 min at physiological temperatures, and the storage modulus (G′) is often within the range of 100–500 Pa, depending on polymer concentration and crosslinking density [[46], [47], [48], [49]]. This mechanical property is essential for ensuring the structural integrity of hydrogels during *in vivo* applications. The degradation rate of pNIPAAm hydrogels when implanted *in vivo* typically ranges from 2 to 4 weeks, which can vary based on the specific formulation used. Additionally, the drug release kinetics of these hydrogels are temperature dependent. For instance, the release half-life of common chemotherapy drugs such as Doxorubicin (DOX) typically spans 12–48 h, controlled by the gelation temperature [50,51]. Hydrogen bonding also plays a pivotal role in physically crosslinked embolic hydrogels, balancing mechanical strength and injectability-key for effective embolization [9,52]. The stable three-dimensional network formed under physiological conditions allows these hydrogels to resist shear stress during injection and maintain structural integrity once embolized. Additionally, hydrogen bonds influence swelling behavior and drug loading capacity, further optimizing the precision of drug release within embolized tumor tissues.

Hydrophobic interaction is one of the most commonly employed and highly reversible mechanisms for constructing injectable, physically cross-linked hydrogels [53]. This mechanism involves incorporating hydrophobic groups into the polymer chain. As hydrophobic groups aggregate in an aqueous environment, the contact area with water decreases, leading to the formation of a stable three-dimensional network structure. This makes hydrophobic interaction particularly suitable for developing thermosensitive, self-healing, injectable, or reversible colloidal systems. These hydrogels show great potential in tumor interventional therapy, tissue engineering, and drug-controlled release [35]. In specific applications, researchers have developed various embolic hydrogel systems based on hydrophobic interactions. For instance, Chen et al. [54] developed an injectable thermosensitive hydrogel using a PLGA-PEG-PLGA block copolymer for the local co-delivery of PLK1 shRNA/polylysine-modified polyethyleneimine (PEI-Lys) complexes and DOX. The hydrogel utilizes hydrophobic aggregation of PLGA blocks and micelle reconstruction induced by partial dehydration of PEG, enabling stable co-delivery and sustained drug release. This thermosensitive hydrogel exhibits a gelation time of approximately 10 min at body temperature and degrades gradually in the subcutaneous layer over a 4-week period, completely disappearing after 5 weeks. Furthermore, the release of DOX at the tumor site follows a sustained release pattern with a half-life of approximately 72 h, enhancing the anti-tumor effects through prolonged drug delivery [55]. Similarly, Jiang et al. [56] developed an intelligent thermosensitive hydrogel constructed via hydrophobic interactions between hydroxybutyl chitosan (HBC) and crocin-1 (CRO). This system responds to temperature changes, achieving rapid gelation at body temperature while maintaining injectability and gel stability, making it suitable for in situ gelation in tumor interventional therapy. In summary, hydrophobic interactions not only provide excellent physical cross-linking properties but, when combined with thermosensitive responsiveness, enhance injectability, controlled release, and targeting in embolization therapy. Hydrophobic interactions significantly enhance the stability and thermal responsiveness of embolic hydrogels, enabling rapid gelation at body temperature [57]. This gelation process ensures hydrogel stability in the vascular environment and sustained drug release at the embolization site. The integration of hydrophobic interactions with thermosensitivity also facilitates self-healing, improving embolization stability and preventing premature degradation.

Ionic/electrostatic interactions are a key physical cross-linking mechanism in hydrogel construction, forming stable three-dimensional networks through electrostatic attraction between polymer chains and multivalent metal ions [58,59]. This mechanism is commonly used in natural polysaccharide-based hydrogels, enabling rapid, reversible gelation under mild conditions while ensuring excellent biocompatibility and injectability [60]. Common materials for ionic/electrostatic cross-linking include alginate, chitosan, and gelatin. Upon exposure to multivalent ions like calcium (Ca^2+^) and magnesium (Mg^2+^), these polymers form a spatial network through ionic bonding, ensuring in situ gelation and structural stability [61,62]. To enhance embolization efficacy and enable multifunctional treatments, various ion-crosslinked hydrogel systems have been developed. For example, Duan et al. [63] developed a thermosensitive composite hydrogel (PSHI-Ca^2+^) by combining poloxamer 407, sodium alginate, and hydroxymethyl cellulose, incorporating Ca^2+^ for transcatheter arterial embolization (TAE) of HCC. This system exhibits temperature-controlled gelation, providing excellent injectability and targeted embolization. The gelation time of the solution, containing 0.5 % sodium alginate, decreased from 110 s at 18 % w/v poloxamer 407 concentrations to 80 s at 22 % w/v. As the temperature increased, the shear stress of the PSHI sample rose from 0.5 Pa to 200 Pa, reaching equilibrium at 32 °C, while the viscosity increased from approximately 0.2 to 3000 Pa s. The PSHI solution demonstrated favorable flowability at room temperature and a drug release half-life of 48 h, showcasing ideal controlled drug release properties for TAE treatment. Lu et al. [64] developed an injectable magnetic colloidal gel (MCG) based on electrostatic attraction between magnetic montmorillonite and amphoteric gelatin nanoparticles, demonstrating synergistic effects in liver tumor models with targeted embolization and magnetic hyperthermia therapy (MHT). Oklu et al. [65] designed a shear-thinning hydrogel with silk protein and nanoclay (NC), maintaining stability under shear stress, suitable for microcatheter injection and intravascular gelation. Similarly, Peng et al. [66] developed a shear-thinning QCS/GA composite gel, showing strong potential for transarterial chemoembolization (TACE) due to its low injection resistance and embolization capabilities. Guo et al. [67] further advanced this technology by creating a degradable ion-exchange platform with grafted nano-polyacrylic acid, which efficiently loads DOX for sustained release at the tumor site. Oklu's team [68] also developed an STB system using porcine gelatin and Laponite XLG, achieving rapid and stable embolic occlusion via electrostatic effects. Lastly, Peng et al. [69] created an injectable composite hydrogel (QTI) with quaternized chitosan (QCS), tannic acid, and iohexol, demonstrating excellent radiographic and embolic capabilities after Na^+^-induced coordination enhanced cross-linking density. In summary, injectable embolic hydrogels based on ionic/electrostatic interactions have emerged as a promising approach in tumor embolization therapy, offering advantages like rapid in situ gelation, structural stability, and broad material sources. These hydrogels are highly promising for clinical tumor embolization applications. Ionic and electrostatic interactions enhance the mechanical strength and stability of embolic hydrogels, which are critical for effective occlusion and drug retention. The formation of stable ionic crosslinks under physiological conditions allows the hydrogel to withstand blood flow forces while maintaining structural integrity. Additionally, ionic crosslinking enables the incorporation of bioactive molecules, enhancing the therapeutic effect through controlled, sustained drug release at the embolization site.

### 2.2 Chemical crosslinking

Injectable hydrogels prepared via chemical crosslinking are formed through covalent bond formation between polymer chains. These reactions can be broadly classified into irreversible (permanent) covalent crosslinking and dynamic (reversible) covalent crosslinking, in addition to photoinitiated and radiation-induced processes. Each bonding mechanism profoundly affects the microstructure, mechanics, and degradation of hydrogels, thereby dictating their performance in tumor embolization and combined therapeutic applications.

**Fig. 3:**
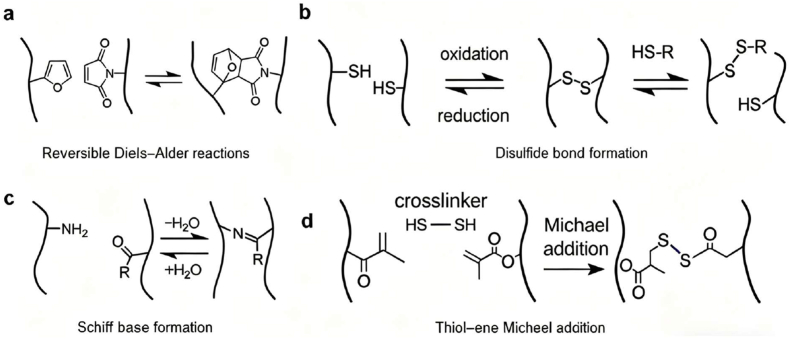
Schematic illustration of representative chemical crosslinking mechanisms in hydrogel embolic agents. (a) Reversible Diels-Alder reactions between furan and maleimide groups form thermally reversible covalent linkages, enabling tunable and self-healing hydrogel behavior. (b) Disulfide bond formation involves the reversible oxidation and reduction of thiol groups, providing redox-responsive crosslinking that enables degradation or reconstruction under physiological redox conditions. (c) Schiff base formation (imine bonding) occurs via condensation between amine and aldehyde groups with reversible hydrolysis, offering pH-sensitive and dynamic covalent crosslinking. (d) Thiol-ene Michael addition provides stable thioether linkages through the nucleophilic addition of thiols to activated alkenes, yielding robust and biocompatible crosslinked hydrogel networks. Together, these representative mechanisms illustrate the chemical versatility available for engineering hydrogel-based embolic systems with controllable degradability, mechanical strength, and therapeutic responsiveness.

Chemical crosslinking can occur through a variety of covalent reactions. For instance, thiol-ene Michael addition provides a classical example of stable, irreversible covalent bonding. In this reaction, thiol groups react with activated alkenes through nucleophilic addition to form thioether linkages, leading to robust, biocompatible hydrogel networks with high structural integrity and chemical stability. In contrast, other reactions such as Diels-Alder cycloaddition, disulfide bond exchange, and Schiff base (imine) condensation introduce reversible characteristics that enable adaptability and self-healing under physiological conditions (Fig. 3).

#### 2.2.1 Dynamic covalent crosslinking

Dynamic covalent chemistry introduces reversibility and responsiveness into hydrogel networks. Bonds can form and break reversibly under specific stimuli such as temperature, redox potential, or pH, enabling self-healing, stress relaxation, and controlled degradation-properties particularly useful for minimally invasive embolic applications.

One representative mechanism is the reversible Diels-Alder reaction (Fig. 3a) between furan and maleimide groups, which forms covalent linkages that can thermally dissociate and reform, granting tunable and thermally reversible gel behavior. Similarly, disulfide bond exchange (Fig. 3b) allows redox-responsive crosslinking through the reversible oxidation and reduction of thiols, making hydrogels sensitive to intracellular or extracellular redox environments. Another widely applied route is Schiff base formation (Fig. 3c), in which imine bonds are generated through condensation between amine and aldehyde groups; their hydrolysis under acidic conditions allows pH-responsive reversibility.

Qu et al. [70] designed a borate-imine dual dynamic crosslinked composite double-network (CDN) hydrogel formed between PVA and CMC, exhibiting rapid sol-gel transition (<1 min), enhanced shear modulus, and adjustable injectability suitable for controlled embolization. Liang et al. [28] further constructed a chitosan-based dynamic self-healing hydrogel using reversible imine chemistry, which demonstrated fast gelation and excellent self-repairing under physiological conditions. Collectively, these dynamic systems embody the adaptability of reversible chemistries to achieve self-healing, on-demand degradation, and mechanical tunability required for precise embolic therapy.

#### 2.2.2 Irreversible covalent crosslinking

Irreversible covalent reactions-such as free-radical polymerization, amidation/imidization, and certain “click” reactions-produce hydrogel networks with strong, permanent bonds and long-term mechanical stability. These materials are particularly advantageous in embolic therapy where durable occlusion and sustained mechanical resistance are essential.

A typical example involves the thiol-ene Michael addition reaction (Fig. 3d), where thiol groups react with electron-deficient alkenes to form thioether linkages. The reaction proceeds efficiently under mild conditions without generating toxic byproducts, producing stable networks with excellent injectability and biocompatibility. Gong et al. [71] developed a double-crosslinked-network (DCN) hydrogel by grafting methacrylic acid groups onto alginate (Alg-MA) and carboxymethyl chitosan (CMC-MA) and employing thiol-ene chemistry (using DTT and TCEP) before injection. The DCN hydrogel achieved a Young's modulus of approximately 5.7 kPa and elongation at break of 234 %, with injection forces manageable through clinical catheters. A secondary in situ thiol-ene reaction under physiological conditions further reinforced mechanical robustness and chemical stability, underscoring its potential for long-term embolization performance.

#### 2.2.3 Photoinitiated (light-activated) chemical crosslinking

Photoinitiated crosslinking (photocrosslinking or photopolymerization) generates reactive species (e.g., radicals) upon light exposure and thereby effects covalent network formation; accordingly, it should be regarded as a subset of chemical crosslinking rather than a separate, parallel class. Photocrosslinking provides exceptional spatial and temporal control over gelation, enabling rapid in situ curing, localized patterning and facile fabrication of microstructured embolic formats [72,73]. UV-initiated free-radical polymerization (200–400 nm) is widely used for hydrogel synthesis under mild aqueous conditions, but is limited by shallow tissue penetration and potential phototoxicity associated with many photoinitiators [[74], [75], [76]]. To mitigate these limitations, visible-light photoinitiators, two-photon activation and NIR-assisted strategies have been developed; these approaches improve penetration depth or reduce photodamage at the cost of tradeoffs in cost, initiator selection and cure efficiency [[77], [78], [79]]. Clinical translation of photocrosslinking therefore requires careful optimization of light dose, photoinitiator biocompatibility and penetration depth.

Photoinitiated chemistries have been successfully applied to fabricate embolic hydrogels and hydrogel microspheres. Zhong et al. combined microfluidic droplet templating with UV-initiated free-radical polymerization to prepare 3Asphere microspheres that degrade in the presence of hyaluronidase (HAase) and display favorable catheter delivery properties (low viscosity 20–1400 mPa s, shear-thinning behaviour and low storage modulus G′ ≈ 90–100 Pa), high drug-loading capacity (EPI up to 100 mg/mL) and sustained release (>95 % EPI over 56 days) [80]. Wang et al. used methacrylated hyaluronic acid and UV crosslinking (365 nm) to produce biodegradable hydrogel microspheres (HAMS) suitable for radionuclide delivery [81], and Liu et al. integrated microfluidics with UV crosslinking to fabricate a lutetium-177 labeled hollow porous particle hydrogel (^177^Lu-3D-HPGH) with high radiochemical stability and biocompatibility [82]. While these examples demonstrate the utility of photoinitiated crosslinking for embolic formulations and microsphere fabrication, careful control of irradiation parameters and initiator chemistry is essential for safe *in vivo* application.

Beyond photoinitiated systems, other energy-driven chemical processes such as radiation-induced crosslinking also enable covalent network formation through reactive species generated by high-energy irradiation. Although differing in energy source and penetration depth, both strategies rely on chemically induced bond formation without the need for additional crosslinkers, providing complementary routes for hydrogel synthesis.

#### 2.2.4 Radiation-induced chemical crosslinking

Radiation-induced chemical crosslinking represents a distinct subclass of chemical crosslinking, wherein ionizing radiation (such as γ-rays or electron beams) generates reactive species-primarily free radicals-that initiate covalent bond formation between polymer chains. Unlike traditional chemical methods that require added initiators or crosslinking agents, radiation crosslinking proceeds without additional reagents, thereby minimizing potential toxicity and ensuring excellent biosafety for biomedical applications [83].

The degree of crosslinking and resultant network properties can be precisely modulated by adjusting the radiation dose, enabling fine control over the hydrogel's mechanical strength, swelling behavior, and degradation profile [84]. Owing to these advantages, radiation-induced crosslinking has become a simple, efficient, and environmentally friendly strategy for hydrogel synthesis [85]. Among ionizing radiation techniques, γ-irradiation is particularly advantageous for graft polymerization and crosslinking since it can be applied to materials in various states (solid or solution) and does not require initiators or catalysts [86]. This characteristic eliminates residual chemical contaminants and supports flexible processing. γ-rays are thus regarded as one of the most effective and sustainable crosslinking approaches [87]. Similarly, electron beam (e-beam) irradiation induces polymerization through high-energy electrons that initiate free-radical reactions in the absence of solvents or initiators. This process simultaneously achieves sterilization, further enhancing its clinical practicality. However, the technique requires high initial investment in irradiation facilities and compliance with strict radiation safety regulations. Radiation-induced crosslinking has been extensively applied in biomedical hydrogels. For example, Liu et al. [88] synthesized polyvinylbenzene microspheres (PEBMs) via emulsion suspension polymerization and grafted 2-methacryloyloxyethyl phosphorylcholine (MPC) monomers through cobalt-60 radiation-induced polymerization to form ^177^Lu-PCMs, which exhibited excellent physicochemical stability, strong hydrophilicity, and dispersibility. The density of PCM (1.11 g/mL) closely matched that of blood (1.05 g/mL), ensuring uniform suspension and distribution. Molecular dynamics simulations revealed favorable interaction energies (van der Waals 0.0205 kcal/mol, electrostatic −0.0011 kcal/mol) and hydrogen bonding (average 4.528 per unit), contributing to the radiostability of coordination structures. In another study, Hegazy [89] employed γ-irradiation to crosslink two anionic polymers-chitosan (Cs) and acrylic acid-co-2-acrylamido-2-methylpropane-sulfonic acid (AAc/AMPS)-to form an amphiphilic Cs/AAc/AMPS hydrogel containing abundant hydrophilic groups, suitable for drug delivery applications. Mohamed et al. [90] developed a pH-sensitive nanocomposite hydrogel via radiation-induced copolymerization of magnesium oxide (MgO), xanthan gum (Xan), and acrylic acid (AAc), achieving controlled methotrexate release at tumor sites. The gelation degree of Xan-AAc increased with irradiation dose, reaching 97 % at 30 kGy, while the swelling ratio (600–1800 %) decreased with higher slurry concentration and dose, consistent with radiation-driven network densification. Jamil [91] prepared an injectable hydrogel via radiation crosslinking of low-molecular-weight chitosan (CS) and PVA, overcoming solubility and viscosity limitations of high-MW chitosan and enhancing injectability and biocompatibility. Popović et al. [92] irradiated natural chitosan (nd-CS) with γ-rays and crosslinked it with PVA doped with silver nanoparticles (Ag NPs), yielding a hydrogel with enhanced mechanical performance and antimicrobial properties.

In summary, radiation-induced chemical crosslinking offers a versatile and reagent-free approach for fabricating biocompatible hydrogels with tunable mechanical and physicochemical characteristics. Its dual functionality in material synthesis and sterilization further supports its potential in drug delivery, tissue repair, and embolization therapies. Continued optimization of radiation protocols and equipment accessibility will further expand the clinical translation of radiation-crosslinked hydrogels.

The distinctions among irreversible, dynamic, photoinitiated, and radiation-induced chemical strategies directly affect injectability, mechanical stability, degradation kinetics, and opportunities for functionalization. Irreversible covalent networks favor long-term permanence and robust mechanical performance; dynamic covalent networks offer adaptive mechanics and self-healing; photoinitiated and radiation-induced approaches both enable externally triggered, controllable in situ curing, with radiation-based methods further offering reagent-free synthesis and inherent sterilization capability. In practice, hybrid strategies (e.g., physical/chemical double networks, dual dynamic/irreversible crosslinking, or photoinitiated secondary curing) are frequently employed to balance deliverability, performance, and safety, as reflected in the embolic systems discussed above.

### 2.3 Hydrogel microspheres synthesis

Hydrogel microspheres are an important class of embolic agents that complement bulk hydrogels by offering improved particle-level control and catheter delivery performance. Microspheres are typically produced by approaches that afford control over size, dispersity, composition and internal structure; commonly used techniques include emulsion polymerization and droplet-based microfluidic templating, as well as spray/electrospray and template-assisted methods.

In emulsion polymerization (conventional or inverse), monomer or prepolymer droplets dispersed in an immiscible continuous phase are polymerized/crosslinked to yield microspheres. This method is scalable and widely employed for synthetic polymers (e.g., acrylates, methacrylates) and for forming crosslinked networks after in-droplet initiation [93]. Microfluidic droplet templating-including flow-focusing and T-junction devices-produces highly monodisperse droplets whose size is tuned by flow rates and channel geometry; droplets can be subsequently crosslinked (ionically, photochemically, or thermally) to form uniform hydrogel beads with narrow size distributions [94]. Coaxial electrospray and spray-drying offer alternative high-throughput routes for forming microspheres from viscous prepolymers or polymer solutions, though they generally yield broader size distributions than microfluidics. Template-assisted and photolithographic methods allow for the fabrication of non-spherical or structured particles, such as anisotropic shapes, for specialized applications (e.g., embolization performance affected by shape). For example, studies have used microfluidics to produce shape-anisotropic PVA-based microspheres for vascular embolization, showing that particle shape impacts navigation ability, distal penetration depth, and stability [95].

Choice of crosslinking chemistry strongly influences microsphere performance. Ionically crosslinked alginate beads are easily formed by dripping into Ca^2+^ solutions and provide rapid, mild fabrication with tunable swelling [96,97]; supramolecular and dynamic covalent chemistries allow post-fabrication injectability and stimuli-responsiveness [98,99]; covalent photopolymerization (e.g., acrylate-based systems) yields mechanically robust beads but often requires careful control of precursor viscosity or in-droplet polymerization to permit catheter delivery [100,101].

The microsphere format offers several advantages as embolic agents: (i) uniform size and narrow dispersity improve predictability of distal lodging and vascular occlusion; (ii) controlled size ranges (from tens to several hundred micrometres) allow tailoring to vessel calibers and catheter navigability; (iii) enhanced surface-area-to-volume ratio facilitates drug loading and release tuning; and (iv) modular composition enables incorporation of imaging tracers (radiopaque/CT-or MRI-visible agents), degradability profiles, or surface ligands for targeting. Design considerations include mechanical robustness to resist fragmentation or compression during catheter delivery, avoidance of particle aggregation or sticking, sterility and endotoxin control, and ensuring predictable *in vivo* swelling and degradation to prevent off-target embolization.

Despite these advantages, challenges remain: achieving both high-throughput production and tight size control (microfluidics vs. scale), balancing mechanical strength with biodegradability, and standardizing particle characterization (size, compressibility, elution profiles) relevant to regulatory approval. Future directions include continuous-flow microfluidic scale-up, hybrid particles with core–shell architectures for staged release or imaging, and rational design of surface chemistry to reduce aggregation and tune biointeractions for safer, more effective embolization.

## 3 Embolic hydrogels for tumor therapy and synergistic treatment strategies

### 3.1 Basic principles of tumor vascular embolization therapy

Malignant tumors, such as HCC, colorectal, prostate, and renal cancers, are typically characterized by a rich blood supply, which contributes to their malignancy, invasiveness, and increased lethality during progression [6]. As a result, traditional treatments like surgery, radiotherapy, and chemotherapy often have limited effectiveness [102,103]. In contrast, TAE has emerged as a promising local treatment. Pioneered by Robert in 1904, TAE has significantly evolved with advances in imaging, enabling precise control during the procedure [104,105]. The development of microcatheters allows for targeted delivery of embolic agents and therapeutic drugs directly to the tumor's blood supply, making TAE a key technique in modern tumor therapy [106,107]. The therapeutic mechanism of TAE is twofold: first, embolization occludes the tumor's blood supply, inducing ischemia and necrosis, thus achieving “starvation therapy” [9]; second, embolic agents facilitate targeted delivery of therapeutic drugs, such as chemotherapy, immunotherapy, and radiotherapy agents, directly to the tumor. This dual approach combines multiple therapeutic strategies, significantly enhancing treatment effectiveness [108,109].

Embolic hydrogels are becoming increasingly important in tumor embolization, offering advantages over traditional embolic materials. Due to their unique physical and chemical properties, hydrogels interact with tumor blood vessels, blood flow, and the microenvironment to enhance embolization efficacy [39,74]. They undergo gelation in response to environmental stimuli (e.g., pH, temperature, ionic strength), ensuring in situ gelation after injection into the tumor vasculature. This controlled gelation minimizes non-target embolization risks [[110], [111], [112]]. Additionally, hydrogels' shear-thinning behavior allows for easy injection through microcatheters, forming stable embolic plugs that block blood supply and induce ischemia. Besides embolization, hydrogels can incorporate therapeutic agents, such as chemotherapeutics, immunotherapeutics, and gene therapies [9,110]. The hydrogel matrix serves as a drug carrier, enabling controlled, localized release at the tumor site, enhancing therapeutic effects. This dual function-embolization and drug delivery-improves TAE efficacy, offering a more targeted approach to tumor treatment.

The interaction between hydrogels and the TME is critical for therapeutic success. For example, hydrogels can respond to the tumor's acidic environment, swelling or degrading to release drugs precisely where needed [113]. Their biocompatibility, coupled with the ability to modulate bioactive molecule release, enables a synergistic effect, improving the efficacy of embolization, chemotherapy, and immunotherapy. Embolic hydrogels enhance tumor embolization therapies by providing both physical blockage and controlled drug delivery. These hydrogels interact with the TME, blood flow, and vasculature to ensure precise embolization. Their ability to incorporate therapeutic agents further increases their effectiveness, making them a promising tool for synergistic tumor treatments.

### 3.2 Therapeutic payload loading and release mechanisms in embolic hydrogels

Beyond their embolic function, hydrogels act as versatile carriers for diverse therapeutic payloads, enabling controlled and localized drug delivery within tumor vasculature. The strategies used for drug loading and release in embolic hydrogels are critical for achieving synergistic therapeutic effects and minimizing systemic toxicity [9,110]. Typically, therapeutic agents such as chemotherapeutic drugs, immunomodulators, radiosensitizers, or gene vectors are incorporated into hydrogels through one or more of the following approaches.

Physical entrapment is the most commonly used loading strategy, where small-molecule drugs (e.g., DOX, cisplatin, 5-fluorouracil) or nanoparticles are mixed with the hydrogel precursors prior to gelation [[114], [115], [116]]. Upon injection, the in sol-gel transition immobilizes the drug within the hydrogel network, forming a depot for sustained release. This method is advantageous for its simplicity and ability to preserve drug bioactivity, making it widely applicable in thermosensitive, pH-responsive, or ionically crosslinked systems [9,61,115].

For drugs or biomolecules requiring higher stability or targeted release, covalent or dynamic chemical bonds are introduced between the hydrogel matrix and the therapeutic molecule. Typical linkages include Schiff-base bonds, disulfide bonds, and ester or amide linkages, which can be cleaved under TME conditions such as low pH or high glutathione concentration [113]. This chemical modification not only prolongs retention but also enables stimuli-responsive release at the tumor site, improving treatment precision.

Incorporating nanocarriers-such as liposomes, micelles, mesoporous silica nanoparticles, or metal-organic frameworks-within hydrogels allows for co-delivery of multiple drugs or the integration of imaging and therapeutic functions [20,21]. These hybrid systems combine the injectability and embolic capacity of hydrogels with the tunable loading and release characteristics of nanocarriers, supporting multimodal treatments including chemo-photothermal and chemo-radiotherapy [107,117,118].

The drug release mechanisms in embolic hydrogels generally depend on both the structural properties of the hydrogel and the physiological environment of the embolized vessel. Diffusion-controlled release occurs through the porous network as small molecules migrate outward with the degradation of the hydrogel matrix. Stimuli-responsive release is triggered by environmental cues such as acidic pH, temperature change, oxidative stress, or enzymatic activity in the TME, allowing for spatiotemporal control of therapeutic delivery [84,[119], [120], [121]]. Degradation-mediated release relies on the hydrogel's intrinsic biodegradability; as hydrolysis or enzymatic cleavage proceeds, entrapped drugs are gradually liberated in a sustained manner. External-field-triggered release, such as photothermal, magnetic, or ultrasound stimulation, has also been integrated into advanced embolic systems to achieve on-demand release and synergistic therapy [64,121,122].

Overall, these strategies enable embolic hydrogels to serve not only as physical occlusion materials but also as intelligent drug delivery platforms capable of precise, controllable, and multifunctional treatment. Such designs effectively combine vascular blockade with local therapy, thereby enhancing the therapeutic efficacy of TAE and its derivatives, including TACE and radioembolization (TARE) [6,52,88].

### 3.3 Application of embolic hydrogels in different tumor types

Embolic hydrogels are delivered directly into the tumor's vasculature via catheter, occluding blood flow to enhance treatment efficacy. These hydrogels, with high-water content, viscoelastic structure, and reversible gelation, transition from liquid to solid at the tumor site, enabling controlled drug release. Their injectable nature and ability to deliver therapeutic agents locally make them well-suited for embolization, particularly when formulated into microspheres for precise delivery to tumors with complex vascular networks. The following sections highlight how hydrogel embolization is adapted to the unique vascular and microenvironmental characteristics of different tumors. To emphasize tumor-specific embolization strategies, we summarize the key microenvironmental features and corresponding hydrogel design considerations in Table 2.

**Table 2:** Comparative characteristics of tumor microenvironments and hydrogel design criteria for embolization.

Tumor Type	Key Microenvironmental Features	Design Criteria for Hydrogel Embolics
Hepatocellular carcinoma	Dual blood supply (hepatic artery + portal vein); high perfusion; mild hypoxia	Arterial-selective, radiopaque, thermoresponsive or in situ–crosslinkable hydrogels for controlled local release and imaging guidance
Renal cell carcinoma	Severe hypoxia; high VEGF expression; acidic microenvironment (pH 6.5–6.8)	Oxygen-releasing or pH/lactate-responsive hydrogels to relieve hypoxia and modulate angiogenesis
Lung cancer	Fragile pulmonary vasculature; high risk of hemoptysis; multiple collateral vessels	Soft, deformable microsphere hydrogels enabling distal, selective embolization and hemostasis with minimal vessel injury
Gynecological tumors (e.g., uterine fibroids)	Hypervascularization; hormone-sensitive growth; fertility preservation desired	Elastic, biocompatible hydrogel microspheres with adjustable swelling, degradability, and high navigability

#### 3.3.1 Embolization therapy for HCC and other liver tumors

HCC and other liver malignancies often present at advanced stages, making surgical resection difficult or impossible for many patients [3,7,59]. The liver's dual blood supply-from the portal vein and hepatic artery-forms the physiological basis for TAE and TACE [123]. In HCC, tumor tissue derives most of its blood supply from the hepatic artery, while healthy liver tissue depends largely on the portal vein. This allows arterial-selective embolization, which can occlude tumor vessels while sparing normal parenchyma. Traditional iodized oil-based TACE suffers from rapid drug release and uneven drug distribution [124,125]. Thermosensitive or in situ-crosslinkable hydrogels have been explored as next-generation embolic agents to overcome these limitations. Hydrogels loaded with iodized oil or chemotherapeutics can undergo sol-gel transition at body temperature, enabling stable and localized embolization. These systems sustain drug release and reduce systemic toxicity while providing radiopacity for image-guided delivery [4,20,21,126]. Such arterial-selective, image-visible embolic hydrogels hold great promise for precise, durable HCC embolization therapy.

#### 3.3.2 Expanded application of embolization techniques in renal cancer

Renal cell carcinoma is a highly vascular malignancy of the urinary system, with a microenvironment characterized by hypoxia-driven VEGF overexpression and acidic metabolism [127]. These factors promote angiogenesis and therapeutic resistance after embolization. To counteract this, recent hydrogel designs have incorporated oxygen-generating components (e.g., CaO_2_-loaded alginate microspheres) that release oxygen post-embolization, mitigating hypoxia-induced VEGF upregulation and enhancing tumor necrosis. Clinically, renal artery embolization (RAE) is applied when resection is infeasible or as a preoperative adjunct to minimize bleeding [128]. When combined with chemotherapeutic agents (e.g., DOX-loaded thermosensitive hydrogels), TAE can achieve both vessel occlusion and localized, controlled drug delivery with minimal systemic toxicity [[129], [130], [131]]. Non-invasive imaging such as PET/CT or X-ray further assists in real-time treatment monitoring [130]. These strategies emphasize tumor-specific microenvironment targeting, leveraging oxygen-releasing or pH-responsive hydrogels to overcome post-embolic hypoxia and acidic stress typical of Renal cell carcinoma.

#### 3.3.3 Application of embolization technology in lung cancer

Lung cancer remains a leading cause of cancer-related death, and hemoptysis is a common, life-threatening complication [[132], [133], [134]]. Arterial embolization, first applied by Viamonte in 1964, remains an essential intervention for managing tumor-associated bleeding [135]. The pulmonary vasculature features extensive anastomoses and fragile vessels, making the choice of embolic material crucial to prevent non-target embolization. Conventional embolic agents (e.g., PVA particles, gelatin sponges, metal coils) are effective for hemostasis but lack controlled degradability and can cause proximal occlusion, limiting repeatability [[136], [137], [138]]. Hydrogel-based microspheres, in contrast, enable distal and selective occlusion, minimizing vascular injury while allowing drug loading for sustained intratumoral release [139,140]. With ongoing integration into multimodal regimens (chemo-, radio-, and immunoembolization), hydrogel embolics can also serve as drug reservoirs and immune modulators, improving both local control and systemic response [141,142].

#### 3.3.4 Embolization therapy in gynecological tumors

Uterine artery embolization (UAE) is a minimally invasive, uterus-preserving therapy used for uterine fibroids, adenomyosis, and select uterine malignancies [143,144]. By blocking arterial blood supply, UAE induces ischemic necrosis and tumor shrinkage, improving symptoms without surgery [145,146]. In treating uterine fibroids, UAE is particularly valuable as it preserves the uterus and fertility, offering a safer and more effective alternative to more invasive procedures [147,148]. The use of biocompatible hydrogel microspheres (e.g., spherical PVA, triacryloyl gelatin, or PEG-based hydrogels) provides precise, deformable embolization tailored to the uterine vasculature [[149], [150], [151]]. Recent innovations include PEG-based hydro-pearl microspheres with adjustable elasticity, achieving near-complete necrosis of uterine fibroids with minimal recurrence [[152], [153], [154]]. Moreover, combining UAE with regional chemotherapy delivery enhances treatment for gynecologic malignancies such as endometrial or cervical cancers [146,148,155,156]. Ongoing advances in imaging-guided navigation and intelligent robotic systems further enhance real-time precision and fertility preservation outcomes.

Although embolization therapy has been applied across multiple tumor types, its success depends on adapting hydrogel design to tumor-specific physiology and microenvironmental challenges. Future research should focus on integrating responsive hydrogels (oxygen-, pH-, or metabolite-responsive) with precise image-guided delivery to further enhance embolization safety and efficacy.

### 3.4 Synergistic anti-tumor therapy with embolic hydrogels

Embolic hydrogels not only serve as mechanical embolic materials but also as multifunctional therapeutic platforms when combined with other treatment modalities. Their 3D porous network, injectability, and responsiveness to external stimuli such as pH, temperature, light, and magnetic fields enable precise and localized drug release within the TME, reducing systemic toxicity [64,65,68,[157], [158], [159], [160]]. Moreover, their biodegradability and imaging visibility allow for real-time guidance and controlled release, facilitating synergistic integration with chemotherapy, radiotherapy, and hyperthermia. Table 3 summarizes the diverse applications and compositions of embolic hydrogels in these combined strategies.

**Table 3:** Representative hydrogels developed for tumor embolization and multifunctional synergistic therapy.

Type	Materials	Components	Crosslinking	Responsive	Injectable	Loading	Function	Reference
Chemotherapy	IBi-D	NIPAM/MBAM	Physical	Temperature	Yes	DOX/iohexol	TACE/DSA	[51]
	PT/DOX-MS	PVA/HA	Electrostatic	–	Yes	DOX/PT-2385	TACE	[117]
	3Asphere	HA	Photopolymerization	Enzyme	Yes	DOX	TACE	[80]
	IF@Gel	Poloxamer-407	Hydrogen bonding	Temperature	Yes	DOX/Fe_3_O_4_	TACE	[164]
	DESTH hydrogel	Gelatin	Electrostatic	pH	Yes	DOX/aPD-1	TACE/ICIs	[114]
	Pt-P@PND	PNIPAM-*b*-PDMAEA	Electrostatic	Temperature	Yes	Cisplatin	TACE	[115]
Radiotherapy	DPT MPs	PVA	Chemical	–	Yes	^177^Lu	TARE	[168]
	^177^Lu-3D-HPGH	CMC/GelMA	Photopolymerization	–	Yes	^177^Lu	TARE	[82]
	^177^Lu-PCM	PVA	Radiation	–	Yes	^177^Lu	TARE	[88]
	^131^I-labeled chitosan hydrogel	Chitosan	–	–	Yes	^131^I	TARE	[169]
	^131^I-SFMs	Silk fibroin	Chemical	–	Yes	^131^I	TARE	[170]
	^131^I-PDA@PVA MS	PVA	Chemical	–	Yes	^131^I	TARE/PTT	[171]
	HA-MA-BP	HA	Photopolymerization	Enzyme	Yes	^90^Y	TARE	[81]
Thermal therapy	Gel-SA-CuO	Gelatin/NaAlg	Ionic crosslinking	–	Yes	CuO NPs	PTT	[175]
	DOX@Au-MnO-L NPs/F127	Pluronic F127	Physical	Temperature	Yes	DOX/Au-MnO NPs	TACE/PTT	[122]
	Iron foam-agarose gel-drug	Agarose	–	–	Yes	DOX	MHT	[179]
	Magnetic colloidal hydrogel	Gelatin	Electrostatic interactions	pH	Yes	–	MHT	[180]
Robotic therapy	MagLiCA microrobots	NaAlg	Ionic crosslinking	Magnetic	Yes	Lipiodol	TAE	[185]
	Microrobotic system	PLGA/gelatin	–	Magnetic	Yes	DOX/5-Fu	TACE	[187]
	Magnetic lipiodol microdroplets	Gelatin	–	Magnetic	Yes	Lipiodol	TAE	[184]
Modulating TME	SA@CaO_2_ MSs	NaAlg	Ionic crosslinking	–	Yes	CaO_2_	TAE/CT	[188]
	PNDS	PNIPAM-DMA	–	Temperature	Yes	Cu chelating agent	TAE	[189]
	PPP@CD	PEG-PPG-PEG/α-CD	Host-guest	–	Yes	–	Pyroptosis	[197]
	PT/DOX-MS microspheres	PVA/HA	Electrostatic	–	Yes	DOX/PT-2385	Hypoxia	[117]
	PNDM	PNIPAM-co-DMA	Physical	Temperature	Yes	Mn^2+^/atechol	Hypoxia	[111]

#### 3.4.1 Embolic hydrogel-mediated chemotherapy

Chemotherapy remains a cornerstone in the management of advanced malignancies and is widely employed in palliative care, tumor downstaging, and postoperative adjuvant settings. However, conventional systemic chemotherapy is often constrained by limited tumor selectivity and significant off-target toxicity, including bone marrow suppression, gastrointestinal disturbances, and alopecia [118]. Localized intratumoral or intra-arterial chemotherapy using embolic hydrogels represents a transformative advance that mitigates these systemic effects while enhancing therapeutic precision [2]. Compared with traditional embolic agents, hydrogel systems further allow the rational tuning of physicochemical properties-such as gelation time, injectability, viscoelasticity, and degradation rate-parameters that directly influence clinically relevant outcomes including distal penetration, reflux prevention, and recanalization risk.

Hydrogels possess high water content (>90 %), reversible sol-gel transition capability, and shear-thinning behavior that facilitate minimally invasive administration through microcatheters, enabling deep penetration into tumor-feeding arteries prior to solidification [21,123]. Fast yet controllable gelation contributes to preventing reflux and ensuring accurate embolic localization, while enhanced mechanical integrity and slower degradation are associated with prolonged occlusion stability and reduced recanalization. These material-dependent clinical correlations underscore the importance of integrating rheological and physicochemical characterization into embolic hydrogel design.

**Fig. 4:**
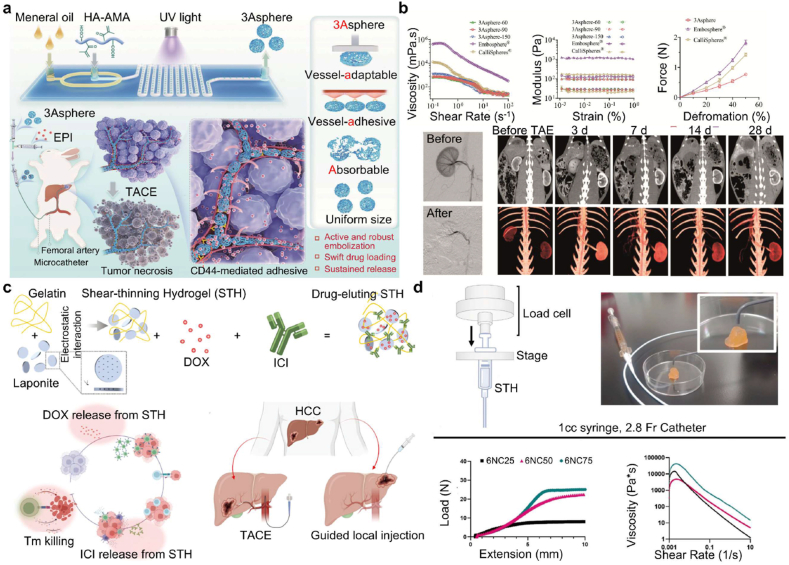
Precise delivery and controlled release of chemotherapy drugs using embolic hydrogels. (a) Uniform hydrogel microspheres loaded with DOX demonstrate effective anti-tumor embolic effects. Reproduced with permission from Ref. [80]. Copyright 2025, Springer Nature. (b) The 3A sphere hydrogel microspheres exhibit advantages such as low viscosity, shear-thinning behavior, and a low storage modulus, which contribute to their stable embolization performance in RAE. Reproduced with permission from Ref. [80]. Copyright 2025, Springer Nature. (c) The DESTH nanocomposite hydrogel, which delivers DOX intratumorally in a pH-responsive manner, has emerged as an effective treatment for HCC. Reproduced with permission from Ref. [114], Copyright 2024, Wiley-VCH. (d) At higher laponite concentrations, the colloidal network of DESTH becomes more rigid. The injection force of 6NC75 ranged from 20 to 25 N. Under shear rates ≥0.1/s, 6NC25 exhibited the lowest viscosity, indicating reduced resistance to flow. Reproduced with permission from Ref. [114], Copyright 2024, Wiley-VCH.

A wide range of hydrogel-based chemotherapeutic platforms has been explored, particularly for DOX, whose systemic applications are limited by severe cardiotoxicity and myelosuppression. Xu et al. [51] developed p(N-isopropylacrylamide-co-butyl methacrylate) nanogels (IBi-D) capable of temperature-responsive DOX release, enhancing intratumoral accumulation while minimizing systemic leakage. Hydrogel microspheres generated via microfluidic technologies further demonstrated excellent monodispersity, high drug-loading efficiency, and sustained release kinetics [161,162]. Ji et al. [117] engineered multifunctional PVA/HA microspheres (PT/DOX-MS) that not only provided stable embolization and DOX release but also induced potent antitumor effects through synchronous G2/M cell-cycle arrest and apoptosis. Similarly, microspheres developed by Zhong and Zhang et al. exhibited prolonged vascular occlusion for up to 28 days in rabbit renal artery models (Fig. 4a and b), highlighting their mechanical robustness and embolization durability [80,163].

Importantly, hydrogel microspheres and bulk hydrogels exhibit distinct advantages and limitations, reflecting intrinsic differences in crosslinking homogeneity, mechanical stability, and drug incorporation modes. Microspheres typically provide predictable embolic behavior, long-lasting occlusion, and near-zero-order drug release due to their uniform internal network structures. Bulk hydrogels or thermosensitive gels, in contrast, offer higher loading capacity and more pronounced stimulus-responsiveness but often present faster burst release or variable long-term release unless reinforced by nanoparticles or dual-crosslinking designs. Therefore, platform selection should consider not only drug properties but also desired embolization depth, vascular morphology, and treatment duration.

Composite hydrogels further expand therapeutic versatility. Li et al. [164] designed an idarubicin-loaded Fe_3_O_4_-poloxamer hybrid hydrogel (IF@Gel) that integrates chemotherapeutic effects with magnetic guidance and hyperthermia-assisted release, enabling precise localization and enhanced cytotoxicity. Kim et al. [114] introduced a gelatin-nanoclay hydrogel (DESTH, Fig. 4c) with pH-responsive DOX release, where increasing nanoclay content (6NC25 to 6NC75) strengthened colloidal interactions and elevated injection force, balancing delivery feasibility with mechanical stability (Fig. 4d). Temperature- and pH-responsive systems such as the thermosensitive cisplatin complex Pt-P@PND [115] and the dual-drug DOX/NCTD hydrogel [112] exemplify how hydrogels exploit local environmental cues-temperature, pH, ionic strength-to achieve on-demand drug release and sustained intratumoral exposure [113].

While applications are often categorized by tumor type, the performance of embolic hydrogels across cancers shares common principles dictated by vascular architecture and TME. Hypervascular tumors such as HCC benefit from stiffer, slower-degrading microspheres that maintain durable occlusion and prolonged drug retention. Hypovascular or heterogeneous tumors may require low-viscosity precursors capable of distal penetration before gelation. Acidic or enzyme-rich microenvironments particularly favor pH-responsive or degradable hydrogels that release drugs in a controlled fashion. These cross-tumor comparisons indicate that optimal therapeutic outcomes arise from the alignment of material properties, drug pharmacokinetics, and tumor-specific vascular physiology, rather than tumor type alone.

By functioning as both embolic agents and controlled drug-release reservoirs, hydrogels significantly enhance intratumoral drug concentration and mitigate systemic toxicity. Their tunable physicochemical characteristics allow precise adjustment of embolization depth, release kinetics, and therapeutic duration, positioning hydrogel-mediated chemotherapy as a central pillar in synergistic hydrogel-based tumor embolization strategies.

#### 3.4.2 Embolic hydrogel-mediated radiotherapy

**Fig. 5:**
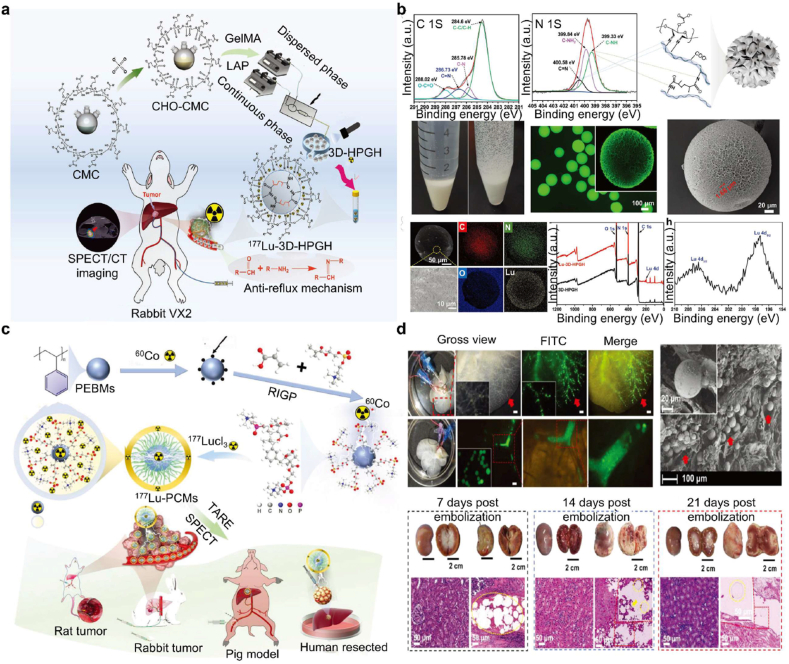
Embolic hydrogels enable precise delivery of radionuclides and play a key role in internal radiotherapy. (a) ^177^Lu-3D-HPGH is delivered to HCC via the hepatic artery, achieving complete tumor suppression at high local radioactive doses. Reproduced with permission from Ref. [82]. Copyright 2023, Wiley-VCH. (b) ^177^Lu-3D-HPGH was prepared using microfluidic technology and UV crosslinking. The particles exhibit excellent mono-dispersity and uniform size in saline. SEM images confirm that 3D-HPGH possesses a three-dimensional hollow porous structure Reproduced with permission from Ref. [82]. Copyright 2023, Wiley-VCH. (c) ^177^Lu-PCM allows for precise delivery of lutetium-177, providing effective anti-cancer treatment. Reproduced with permission from Ref. [88]. Copyright 2023, Wiley-VCH. (d) Local vascular distribution and stable embolization effect of ^177^Lu-PCM in the rat transparent liver and rabbit RAE models. Reproduced with permission from Ref. [88]. Copyright 2023, Wiley-VCH.

Intratumoral radiotherapy delivers radionuclides directly to the tumor site through injection or transcatheter arterial radioembolization, producing anti-tumor effects [165]. This localized method concentrates the radionuclides within the tumor, maximizing their cytotoxic effect while minimizing radiation damage to surrounding healthy tissues [166]. Currently, the most commonly used radionuclides for tumor radiotherapy include ^177^Lu, ^131^I, and ^90^Y. By combining these radionuclides with embolic hydrogels, both the delivery efficiency and stability of radiotherapy within the tumor can be significantly enhanced, improving the overall therapeutic outcome [107]. For example, ^177^Lu releases β^−^ particles that effectively kill tumor cells, while its γ rays enable single-photon emission computed tomography (SPECT) imaging, allowing for integrated tumor diagnosis and treatment. Wang's team [167] developed ^177^Lu-labeled microspheres (^177^Lu-MS@PLGA) by combining poly (lactic-co-glycolic acid) (PLGA) with radioactive silica microspheres. These microspheres demonstrated high radiostability and effectively inhibited tumor growth without significant side effects. Similarly, Song et al. [168] developed traceable PVA microspheres (DPT MPs) labeled with ^177^Lu for close-range treatment. Coupled with SPECT technology, these microspheres facilitated long-term tumor monitoring post-embolization and significantly inhibited tumor growth. A lutetium-177-labeled double-crosslinked hydrogel (^177^Lu-3D-HPGH) was prepared by Liu [82] and precisely delivered to rabbit VX2 liver tumors through the hepatic artery. This approach achieved complete tumor inhibition at a high local radioactive dose, enabling radiotherapy of vascularized tumors under SPECT guidance (Fig. 5a). ^177^Lu-3D-HPGH was prepared using microfluidic technology and UV crosslinking, demonstrating excellent monodispersity in saline for precise radiolabeled hydrogel particle delivery. The average particle size is 150 ± 20 μm, and SEM images reveal a three-dimensional hollow porous structure with a pore size of ∼1.68 μm. The high specific surface area and 3D structure enhance the coordination of C=N groups with Lu^3+^, improving the radiolabeling reaction kinetics for efficient localized radiotherapy (Fig. 5b). In addition, lutetium-177 coordination polymer microspheres (^177^Lu-PCM), modified with phosphorylcholine, exhibited excellent mechanical properties and hydrophilicity (Fig. 5c), allowing for precise lutetium-177 delivery and effective anti-cancer treatment [88]. ^177^Lu-PCM shows good dispersion in the Wistar rat transparent liver vasculature. SEM images confirm that the microspheres retain their spherical shape after injection, freezing, and drying, demonstrating strong stability and compression resistance. In the rabbit renal embolization model, ^177^Lu-PCM maintains effective vascular occlusion for up to 21 days, highlighting its excellent embolization and mechanical stability *in vivo* (Fig. 5d). Similarly, iodine-131 (^131^I) emits beta rays to irradiate tumor cells, producing anti-tumor effects. A^131^I-labeled chitosan hydrogel (Chi) was developed by Jeong and colleagues [169], which showed significant enrichment in the liver and effectively inhibited tumor growth after injection through the hepatic artery. Hydrogel-based systems enhance the efficiency and precision of radioembolization by improving the stability and release kinetics of the radionuclide. The hydrogel matrix serves dual purposes: as an embolic agent to block blood flow to tumors and as a controlled drug delivery system. This synergy improves radiation retention and localized delivery, optimizing treatment outcomes. Zhang et al. [170] prepared ^131^I-labeled silk fibroin microspheres (^131^I-SFMs), which exhibited a labeling rate of over 84 % and excellent radiostability. These microspheres successfully achieved hepatic artery radioembolization and significantly inhibited HCC progression. Wang's team [171] utilized PVA gel microspheres coated with polydopamine (PDA) as a carrier for ^131^I. The resulting ^131^I-PDA@PVA microspheres effectively inhibited tumor growth while avoiding significant side effects when combined with radioembolization and photothermal therapy (PTT). Additionally, Lu and collaborators [172] incorporated tyrosine into PVA microbeads to develop ^131^I-labeled transarterial radioembolization (TARE) drugs. These drugs exhibited high tumor specificity and long-term retention, leading to significant tumor accumulation at low doses and a synergistic effect on the treatment of HCC. Yttrium-90 (^90^Y) emits beta rays to achieve precise and continuous tumor treatment, making it particularly suitable for treating primary HCC and colorectal cancer liver metastases. Wang et al. [81] used biodegradable hyaluronic acid as a scaffold, modified with bisphosphonates and methacrylic acid, to prepare ^90^Y-labeled biodegradable gel microspheres (^90^Y-HAMS). After TAE treatment, the microspheres effectively inhibited the growth of HCC and achieved sustained tumor suppression with minimal side effects. The microspheres exhibited controlled release kinetics, with a drug release half-life of 2.64 days and an embolization duration of up to 14 days, ensuring long-term efficacy. Similarly, Qian et al. [173] developed a new type of ^90^Y-labeled carbon microspheres (^90^Y-HUACM) with high uniformity and specific activity. These microspheres exhibited excellent anti-reflux properties, precise embolization, and biosafety, showing significant tumor inhibition effects. The effectiveness of embolic hydrogels in radiotherapy is attributed to their unique physical and chemical properties, such as shear-thinning behavior, temperature responsiveness, and controlled release mechanisms. Hydrogels respond to environmental stimuli, including pH, temperature, and ionic strength, enabling precise control over drug and radionuclide release [10,107]. In the tumor's acidic microenvironment, hydrogels degrade or swell, releasing therapeutic agents or radionuclides where needed, enhancing overall therapeutic effects [174]. By encapsulating and locally releasing therapeutic agents, hydrogels improve the precision and effectiveness of combined tumor therapies when used with embolization.

#### 3.4.3 Embolic hydrogel-mediated hyperthermia

Hyperthermia is a non-invasive technique used to locally increase the temperature of tumor tissues, thereby effectively killing tumor cells. This approach primarily includes PTT and MHT. In PTT, Li et al. [175] developed a hydrogel scaffold (Gel-SA-CuO) composed of gelatin, sodium alginate, and CuO nanoparticles. After near-infrared laser irradiation, the tumor temperature gradually rose to approximately 48 °C, significantly reducing tumor volume. In another study, a thermosensitive hydrogel (F127) was developed by Zhang and colleagues [122], incorporating DOX and gold manganese oxide (Au-MnO) nanoparticles to form the DOX@Au-MnO-L NPs/F127 hydrogel (DAML/H). This system demonstrated injectable properties and stable photothermal conversion capabilities, allowing for on-demand, sustained-release tumor treatment. Furthermore, Zhu and collaborators [176] applied temperature-adaptive hydrogel fiber optical waveguide (THFOW) technology, which demonstrated excellent affinity for soft tissue, characterized by low cytotoxicity, stability during expansion, and a Young's modulus comparable to that of soft tissue. This hydrogel effectively eliminated deep tumor cells while minimizing the risk of overheating.

**Fig. 6:**
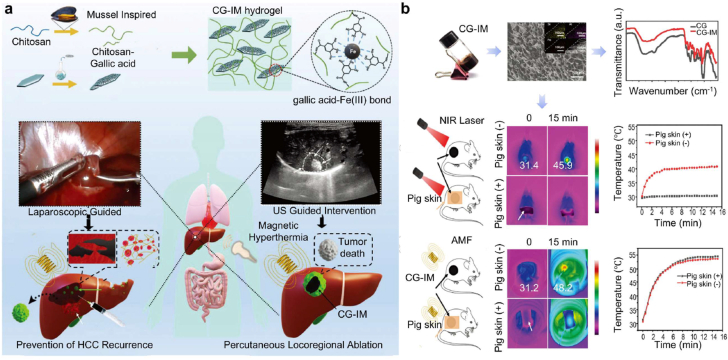
Embolic hydrogels deliver thermoresponsive materials, exerting significant anti-tumor effects within the tumor. (a) CG-IM exhibits excellent magnetothermal properties, providing effective treatment for local ablation of HCC. Reproduced with permission from Ref. [182]. Copyright 2024, Wiley-VCH. (b) The internal structure and composite distribution of CG-IM hydrogel were characterized by scanning electron microscopy and energy dispersive spectroscopy mapping. Subcutaneously injected CG-IM hydrogel demonstrated effective thermotherapy under AMF. Reproduced with permission from Ref. [182]. Copyright 2024, Wiley-VCH.

In MHT, magnetic nanoparticles induce a local temperature rise when exposed to an alternating magnetic field, facilitating tumor treatment [177]. MHT offers excellent penetration ability and anti-tumor effects, making it highly suitable for treating deep tumors, such as those in the liver, brain gliomas, and prostate cancer [178]. Wei et al. [179] applied foam iron-agarose gel-drug (IF-Aga-drug) in the combined treatment of HCC. The foam iron generates substantial heat through the eddy current loss effect in the alternating magnetic field, effectively achieving magnetic hyperthermia. Similarly, a magnetic colloidal hydrogel was constructed by combining superparamagnetic Fe_3_O_4_ nanoparticles with a gelatin nanoparticle system, as described by Yu and colleagues [180]. This system exhibited excellent MHT response properties, continuously generating heat under an alternating magnetic field. Notably, it was not limited by depth penetration, successfully achieving significant therapeutic effects in mouse and rabbit models. In another study, a magnetic hydrogel composed of a triblock polymer matrix and reduced graphene oxide nanosheets loaded with iron oxide nanoparticles (Fe_3_O_4_@rGO, referred to as FG) was reported to exhibit excellent biocompatibility, thermal responsiveness, strong adhesion, and high magnetothermal efficacy [181]. This hydrogel showed promise for postoperative treatment and transarterial embolization in HCC. Additionally, the magnetic nanocomposite hydrogel (CG-IM) loaded with iron oxide nanoparticles demonstrated injectability, magnetothermal properties, mechanical strength, and hemostatic capabilities, effectively performing local ablation treatment for HCC (Fig. 6a) [182]. CG-IM hydrogel exhibits a well-structured gel network, as shown by scanning electron microscopy. Subcutaneously injected CG-IM hydrogel demonstrates strong magnetic and photothermal effects. When covered with pig skin at the injection site, the heating rate and maximum temperature of the magnetic effect remain stable (Fig. 6b). By combining the excellent therapeutic penetration of thermal therapy with the embolization advantages of the hydrogel, targeted thermotherapy can be achieved, significantly reducing thermal damage to normal tissues, thus offering potential for deep tumor treatment.

#### 3.4.4 Hydrogel robot precision treatment

**Fig. 7:**
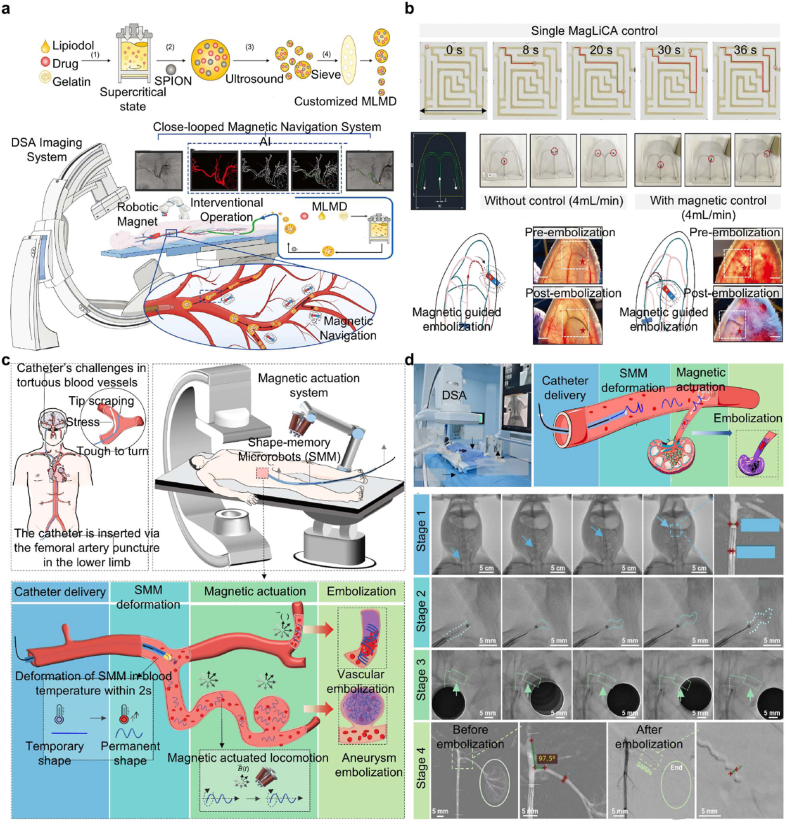
Embolic hydrogel robots achieve intelligent and precise tumor embolization. (a) MLMDs efficiently perform navigation tasks, accurately targeting lesions under the control of an external magnetic field to achieve precise embolization. Reproduced with permission from Ref. [184]. Copyright 2025, Wiley-VCH. (b) MagLiCA microrobots, highly resolvable under X-ray imaging, are expected to enable precise drug delivery and treatment through real-time. Reproduced with permission from Ref. [185]. Copyright 2024, Wiley-VCH. (c) SMMs can overcome narrow and tortuous vascular obstacles under a rotating magnetic field, moving flexibly through blood vessels to achieve multi-level vascular embolization. Reproduced with permission from Ref. [186]. Copyright 2024, American Association for the Advancement of Science. (d) SMMs enable rapid and efficient renal embolization. Reproduced with permission from Ref*.* [186]*.* Copyright 2024, American Association for the Advancement of Science.

Traditional tumor arterial embolization heavily depends on the surgeon's manual skills, making it challenging to ensure accurate embolization in complex vascular environments. Additionally, the high level of clinical experience required means that the training period for skilled interventional doctors is extensive. Moreover, long-term surgeries under X-ray exposure pose a significant health risk to interventional practitioners. The advent of robotic embolization technology offers a promising solution to these challenges. Micro-embolization robots have overcome the limitations of traditional catheters, providing a new approach for precise tumor embolization treatment. During the embolization procedure, robotic systems, through programming and precise navigation, overcome the constraints of traditional surgical techniques. They are capable of reaching narrow and tortuous tumor vessels, enabling precise embolization. Magnetically controlled robots, for example, can intelligently and precisely navigate to target tumor blood vessels under magnetic field control, achieving accurate embolization [183]. For instance, Liu et al. [184] prepared water-stable magnetic iodized oil droplets (MLMDs) using clinically approved iodized oil, gelatin, and superparamagnetic iron oxide nanoparticles (SPIONs). These MLMDs efficiently perform navigation tasks, such as accurately locating target sites, identifying obstacles, following predetermined paths, and planning motion under external magnetic field control. This system allows for precise targeting of lesion areas (Fig. 7a). Liu et al. developed a programmable multifunctional micro-robot using the iGHOST high-throughput oscillatory shear technique. Under the control of a permanent magnet, a single MagLiCA micro-robot completed a maze navigation experiment in approximately 36 s, demonstrating strong magnetic control and flexibility. It achieved precise magnetic targeting embolization in a plastic model with a 2 mm inner diameter and rabbit ear arteries. The MagLiCA micro-robot shows high resolution under X-ray imaging, holding promise for precise drug delivery and therapy under real-time imaging guidance (Fig. 7b) [185]. Xu and collaborators [186] designed a shape-memory magnetic microrobot (SMM) by embedding magnetic particles into an organic gel. This microrobot can flexibly navigate narrow and tortuous blood vessels under a rotating magnetic field, achieving multi-level vascular embolization (Fig. 7c). SMMs achieved complete renal embolization within 2 min, with a total transport distance exceeding 100 cm and a 97.5° turn. The linear SMM inside the catheter was driven by a guidewire at 50 mm/s, 15.7 times faster than the maximum *in vivo* magnetic-driven speed of 3.18 mm/s. This setup allows the catheter to serve as a high-speed pathway for the linear SMMs, ensuring time efficiency (Fig. 7d). In another example, Choi's team [187] created a microrobot capable of real-time X-ray and MRI visualization. This microrobot, composed of a hydrogel-encapsulated porous structure and magnetic nanoparticles, enables targeted delivery of both therapeutic and imaging agents. It overcomes the limitations of existing microrobots in liver chemoembolization.

#### 3.4.5 Hydrogel embolization therapy regulates the TME

The TME comprises a complex and dynamic network of stromal cells, immune components, extracellular matrix, and aberrant vasculature, all of which orchestrate tumor progression, metastasis, and therapeutic resistance. Pathophysiological features such as immune suppression, metabolic reprogramming, and hypoxia hinder the efficacy of traditional therapies. Consequently, hydrogel-based embolization offers a unique means to reshape the TME by modulating oxygenation, pH balance, immune activity, and localized drug distribution.

**Fig. 8:**
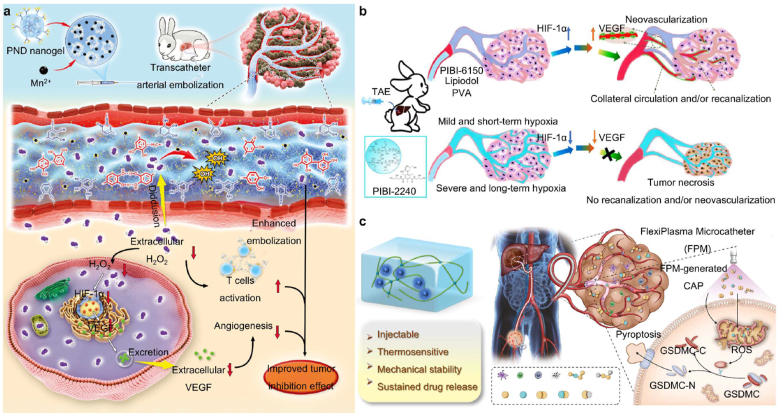
Strategies for regulating the TME using embolic hydrogels. (a) PNDM hydrogel inhibits angiogenesis and tumor metastasis after tumor embolization therapy, significantly prolonging patient survival. The hydrogel delivers therapeutic agents to tumor sites, affecting the tumor vasculature and reducing the spread of cancerous cells. Reproduced with permission from Ref. [111]. Copyright 2022, Wiley-VCH. (b) Regulation of the local hypoxic microenvironment plays a critical role in improving anti-tumor effects. The modulation of oxygen levels within tumors enhances the effectiveness of embolization and promotes the activation of immune responses. Reproduced with permission from Ref. [120]. Copyright 2018, Ivyspring International Publisher. (c) PPP@CD hydrogel enhances the therapeutic effect of HCC through a non-inflammatory cell pyroptosis mechanism. This mechanism induces tumor cell death while minimizing inflammation in the surrounding tissue, improving the overall therapeutic outcome. Reproduced with permission from Ref. [197]. Copyright 2025, Wiley-VCH.

Hydrogels provide sustained and controlled delivery of therapeutic agents directly within tumor tissues, ensuring a prolonged pharmacological effect and a more homogeneous intratumoral distribution. Liu et al. [188] demonstrated that calcium peroxide (CaO_2_) microspheres combined with TAE induced calcium-dependent apoptosis and tumor calcification. In vivo, this approach achieved nearly complete tumor necrosis within ten days, with radiographic evidence of dense embolic regions. Similarly, Zheng et al. [189] formulated a thermosensitive hydrogel (PNDS) incorporating a copper chelator (SO-N), which selectively depleted Cu^2+^ ions, suppressing angiogenesis and enhancing HCC treatment efficacy by restoring copper homeostasis. Beyond ion modulation, tumor metabolism-driven stimuli have emerged as promising design cues for embolic hydrogels. The Warburg effect-characterized by enhanced glycolysis and lactate accumulation even under normoxic conditions-leads to acidification of the TME (pH 6.5–6.8) [190]. This metabolic shift can be exploited for pH-responsive drug release, for instance using imine-linked DOX prodrugs that undergo hydrolysis in acidic conditions to trigger localized chemotherapy while minimizing off-target effects [191]. Moreover, buffering or basic components within hydrogels can partially neutralize local acidity, improving drug diffusion and reducing acidosis-induced immune suppression [192]. In addition to pH-sensitive strategies, reactive oxygen species (ROS)-responsive and ROS-scavenging hydrogels play a dual regulatory role. On one hand, controlled ROS generation can induce tumor cell apoptosis; on the other, excessive ROS may promote inflammation and vascular recanalization after embolization. Hydrogels incorporating thiolated chitosan or phenolic crosslinkers can scavenge excessive ROS, thereby reducing inflammation-driven recanalization, protecting endothelial integrity, and enhancing embolic stability [193]. Such ROS-modulatory systems help maintain a balanced oxidative state conducive to long-term vascular occlusion and local immune activation. Zhang et al. [194] introduced a dual-responsive embolic agent composed of zein, DOX, and mesoporous hollow MnO_2_ (HMnO_2_) nanoparticles. Within the acidic TME, this system generated ROS and released oxygen while decomposing to release DOX, producing synergistic ROS-enhanced TACE effects and alleviating hypoxia. Wang et al. [111] designed a thermosensitive hydrogel (PNDM) that improved animal survival, inhibited angiogenesis, and prevented metastasis following embolization, highlighting hydrogel versatility for microenvironment modulation (Fig. 8a). Hydrogel formulations also modulate intracellular signaling and autophagy pathways. Zhu et al. [195] reported PAA/CaP nanoparticles that increased intracellular Ca^2+^ levels, inhibiting autophagy and enhancing the cytotoxicity of epirubicin. Meanwhile, Ji et al. [117] created PT/DOX-MS microspheres co-loaded with the HIF-2α inhibitor PT-2385, effectively targeting hypoxic HCC cells by downregulating VEGF and improving drug responsiveness under hypoxia. pH modulation remains another vital mechanism. The acidic TME, resulting from elevated glycolysis and lactic acid accumulation, fosters immune evasion and drug resistance. Hydrogels incorporating buffering components or basic nanoparticles can locally neutralize acidity, improving drug diffusion and immune activation. Yang et al. [120] demonstrated that TAE-induced pH changes significantly influenced HCC response (Fig. 8b), emphasizing the need for microenvironmental pH regulation during embolization. Additionally, hydrogels provide a platform for immunomodulation within the TME. Ji et al. [196] utilized platelet membrane-coated nanoparticles co-loaded with erastin and lysyl oxidase (LOX) to enhance ferroptosis and reverse lactate-mediated immune suppression. Liu et al. [197] further demonstrated PPP@CD hydrogels that triggered pyroptosis and released DAMPs, promoting cytotoxic immune infiltration (Fig. 8c).

Collectively, these findings illustrate that hydrogel embolization therapy not only serves as a physical means of vascular occlusion but also acts as a biological modulator of the TME. By integrating oxygen release, ion regulation, metabolic and ROS responsiveness, pH correction, and immune activation, embolic hydrogels foster a microenvironment conducive to therapeutic synergy, bridging embolization with advanced chemo-, radio-, and immunotherapies.

#### 3.4.6 Immunomodulatory hydrogel systems in embolization-based therapy

Immunotherapy has profoundly reshaped cancer treatment; however, its overall efficacy remains constrained by the immunosuppressive tumor immune microenvironment. Hydrogels offer a versatile platform for overcoming these limitations because they can be engineered to provide localized, sustained, and programmable immune modulation. Importantly, immunomodulatory hydrogel systems can act through distinct mechanisms depending on administration route, and conflating these routes may obscure the conceptual differences between intravascular embolization and intratumoral immunotherapy. Therefore, this section reorganizes immunomodulatory hydrogels according to delivery pathway-intra-arterial immunoembolization versus intratumoral hydrogel immunotherapy-to clearly articulate their roles in hydrogel-based tumor embolization and synergistic therapeutic strategies.

**Fig. 9:**
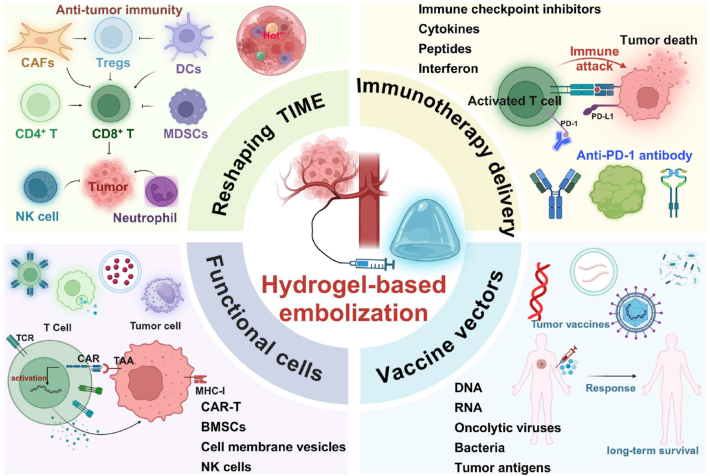
Schematic diagram of the immunomodulatory mechanism of embolic hydrogels. This figure illustrates how hydrogel-based embolization can modulate the immune system to enhance anti-tumor immunity. Key components include: CAF (cancer-associated fibroblasts), tregs (regulatory T cells), CD4^+^ T cells, CD8^+^ T cells, DCs (Dendritic cells), MDSCs cells (Myeloid-derived suppressor cells), NK (Natural Killer) cells and neutrophils, immune checkpoint inhibitors (e.g., anti-PD-1 antibody), tumor vaccines and immunotherapy delivery. Created by BioRender.com.

When delivered through tumor-feeding arteries, hydrogels can function simultaneously as embolic agents and immune modulators, achieving vascular occlusion while enhancing antitumor immune activation. Intra-arterial administration also enables the precise, flow-guided deposition of nanoscale immunomodulators, including nanogels, nanobodies, and cell membrane-derived vesicles, which accumulate within the embolized tumor bed and act as locally retained immune stimulants (Fig. 9). Thus, these nanoscale systems can participate directly in immunoembolization when co-administered or co-formulated with hydrogels.

**Fig. 10:**
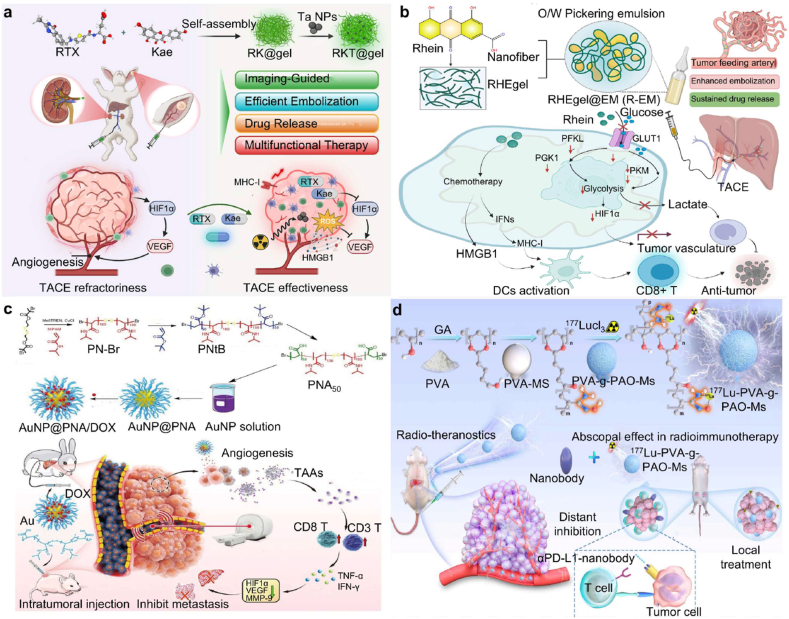
Embolic hydrogels regulate the immune microenvironment of tumors and contribute to embolic immunotherapy. (a) RKT@gel significantly induces ICD, enhancing dendritic cell activation and promoting anti-tumor immune recognition mediated by MHC-I. Reproduced with permission from Ref. [9]. Copyright 2025, Elsevier. (b) Rhein nanogels, developed as an oil-in-water iodized oil embolic emulsion, induce ICD, activate dendritic cells, and present MHC-I to CD8^+^ T cells to effectively kill tumor cells. Reproduced with permission from Ref. [52]. Copyright 2024, Wiley-VCH. (c) AuNP@PNA/DOX enhances the activation of CD3+/CD8+ T cells and their related cytokines in tumors, showing a synergistic effect in improving the tumor immunosuppressive microenvironment. Reproduced with permission from Ref. [200]. Copyright 2023, Springer Nature. (d) The combination of ^177^Lu-PVA-g-PAO-Ms and anti-PD-L1 nanoantibodies offers a novel therapeutic strategy by integrating radioembolization with systemic immunotherapy. Reproduced with permission from Ref. [174]. Copyright 2024, Elsevier.

Hydrogel embolization inherently induces immunogenic cell death (ICD) by triggering ischemia, hypoxia, and metabolic stress. Feng et al. [198] developed thrombin-loaded gelatin microspheres containing CaCO_3_ nanoparticles (Th-CaCO_3_@MSs), which achieved effective embolization, neutralized the tumor acidic microenvironment, and amplified ICD-driven immune activation. Liu et al. [9] reported a self-assembling raltitrexed hydrogel (RKT@Gel) that promoted ICD, dendritic cell maturation, and MHC-I-restricted cytotoxic T cell priming (Fig. 10a). Other intra-arterial immunoembolic platforms integrate metabolic modulation with immune activation. Rhein-nanogel-stabilized iodized oil emulsions enhanced ICD and dendritic cell activation, representing a metabolism-immunity coupled “immunoembolic” strategy (Fig. 10b) [52]. Inorganic nanocomposite hydrogels, such as Fe_3_O_4_-poloxamer (IF@Gel) [164] and selenium nanoparticle hydrogels (PPS-SeNPs@DOX) [199], further boosted ICD signaling and synergized with anti-PD-L1 therapy. Ji et al. [109] created A2AR antagonist/DOX co-loaded gelatin microspheres (SLNP-SCH/DOX@MS), which restored dendritic cell function and reversed T-cell exhaustion. Hydrogels may also couple immunotherapy and radiotherapy within an embolization framework. Liu et al. [174] generated ^177^Lu-labeled PVA microspheres (^177^Lu-PVAg-PAO-Ms) that elicited potent radioimmunotherapeutic effects when combined with anti-PD-L1 nanoantibodies (Fig. 10d). AuNP@PNA hydrogels encapsulating DOX suppressed HIF-1α, VEGF, and MMP-9 while activating CD3^+^/CD8^+^ T cells, reshaping the immunosuppressive tumor immune microenvironment after embolization (Fig. 10c) [200]. Collectively, intra-arterial immunoembolic hydrogels unify vascular targeting, ischemia-induced ICD, and immune activation-constituting a central pillar of hydrogel-based tumor immunoembolization.

In contrast to intra-arterial embolization, intratumoral hydrogels do not occlude vessels. Instead, they act as local immunotherapeutic depots, enabling prolonged release of immune checkpoint inhibitors (ICIs), cytokines, peptides, tumor vaccines, nanogels, nanobodies, or cell membrane vesicles [21,125,178]. Although functionally distinct from embolic hydrogels, intratumoral systems play an important role as adjuncts within embolization-centered treatment regimens-either as preconditioning formulations to sensitize tumors ahead of embolization or as post-embolization boosters enhancing immune infiltration into ischemic tissue. Hydrogels with tailored degradation kinetics can improve intratumoral residence of CAR-T cells, NK cells, or dendritic cells, enabling sustained local immune activation. Nanogel- and vesicle-integrated hydrogels enhance antigen presentation and augment responses to systemic ICIs. Marabelle et al. [201] developed an anti-CTLA-4 Pickering emulsion suitable for both intra-arterial and intratumoral administration, illustrating how the same material platform may exert distinct functions depending on delivery route. Thus, intratumoral hydrogels serve as complementary synergistic tools, expanding the therapeutic impact of embolization by enhancing immune activation within and beyond the ischemic tumor region.

By distinguishing between intra-arterial immunoembolic hydrogels and intratumoral immunotherapeutic hydrogels, it becomes clear how each administration route offers complementary advantages in embolization-centered combination therapy. Intra-arterial hydrogels integrate vascular occlusion with immune activation, whereas intratumoral hydrogels act as potent immunomodulatory adjuncts that enhance the therapeutic impact of embolization. Together, these strategies hold significant promise for converting immune-resistant tumors into responsive phenotypes and advancing synergistic hydrogel-based tumor embolization therapies.

#### 3.4.7 Embolic hydrogels for imaging

Embolic hydrogels are precisely delivered to the tumor site via microcatheter, and the entire embolization process is typically performed under imaging guidance. This allows for monitoring of the embolic agent's delivery to the tumor-feeding artery, controlling the embolic dose, and effectively preventing ectopic embolism. Additionally, post-operative evaluation of the embolic effect is possible. Commonly used interventional imaging methods include non-radioactive embolic agents, MRI embolic agents, ultrasound embolic agents, and radionuclide PET/SPET-CT, all of which are utilized to visualize embolic hydrogels [202].

**Fig. 11:**
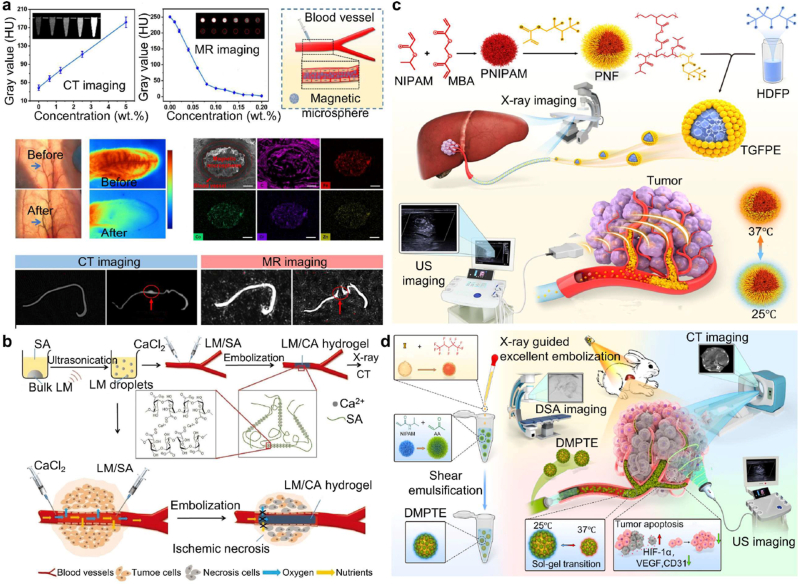
Embolic hydrogel delivery system for real-time imaging. (a) Magnetic hydrogel microspheres release loaded drugs in a controlled manner using alternating magnetic fields, with real-time imaging performed under CT/MRI modalities. Reproduced with permission from Ref. [210]. Copyright 2022, Elsevier. (b) The imaging characteristics of LM/CA hydrogels under X-ray and CT facilitate tracking material positions during vascular surgery. Reproduced with permission from Ref. [211]. Copyright 2020, Wiley-VCH. (c) HDFP Pickering emulsion shows potential for ultrasound-guided embolic materials in interventional TAE procedures. Reproduced with permission from Ref. [121]. Copyright 2023, Springer Nature. (d) Poly (N-isopropylacrylamide-acrylic acid) (PNA) mixed with iodized oil and fluorocarbon compounds enable dual-mode imaging using DSA and ultrasound. Reproduced with permission from Ref. [215]. Copyright 2025, Elsevier.

Radiopaque materials, such as iodized oil, iodine-containing compounds, gold nanoparticles, tantalum nanoparticles, and barium nanoparticles, are commonly used to prepare radiopaque embolic agents [203]. For instance, Cho et al. [204] prepared an embolic hydrogels system using manganese ion-crosslinked HA-dopamine (HD) and iopamidol. This system successfully reached distal arterioles, provided better therapeutic effects, and established a foundation for TACE therapy. Similarly, PVA-2-acrylamido-2-methylpropanesulfonic acid (AMPS) hydrogel microbeads (DC Bead™) were developed by Lewis and colleagues [205], achieved by coupling 2,3,5-triiodobenzaldehyde to the PVA backbone of the microbeads. These microbeads, which can be injected into the hepatic artery under fluoroscopic guidance, can be visualized *in vivo* by CT imaging. The radiocontrast agent barium sulfate (BaSO_4_) has excellent imaging properties [206]. Shen et al. [207] synthesized barium alginate (ALG) microspheres loaded with in situ synthesized barium sulfate (BaSO_4_) using microfluidic droplet technology. These microspheres demonstrated excellent X-ray imaging properties, allowing for direct observation of their position and embolization efficiency after treatment. Guo's team [208] employed droplet microfluidics technology to synthesize CaAlg-IO microbeads, which consisted of cross-linked calcium alginate (CaAlg) and iodized oil droplets. These microbeads exhibited good X-ray imaging properties and excellent ultrasound imaging capabilities, optimizing the evaluation of intravascular embolization therapy. Additionally, Kong et al. [209] prepared sodium hyaluronate (SH) microspheres loaded with bismuth sulfide (Bi_2_S_3_) nanorods (Bi_2_S_3_@SH) through microfluidic technology. These microspheres demonstrated strong X-ray barrier properties and excellent embolization effects and imaging properties. Furthermore, a magnetic microsphere, based on low Curie temperature superparamagnetic iron oxide nanoparticles, was developed by Zhang and collaborators [210]. These microspheres can be controlled to release loaded drugs via an alternating magnetic field and are clearly visible under CT/MRI imaging, making them suitable for TACE treatment (Fig. 11a). Liu et al. [211] prepared an injectable, radiopaque liquid metal/calcium alginate (LM/CA) hydrogel that demonstrated radiopaque properties under X-ray and CT scans, significantly improving the tracking of material position during vascular surgery (Fig. 11b).

Despite their effectiveness, X-ray imaging carries a risk of radiation exposure, which can be harmful to interventional surgeons with long-term exposure. Therefore, the development of new, safe, and radiation-free embolic materials is a promising area of research. MRI has become a potential alternative imaging method due to its excellent imaging capabilities and safety profile. Kim and his team created embolic microspheres composed of calcium alginate and ultra-small superparamagnetic iron oxide nanoclusters. The T2-weighted MRI characteristics of these microspheres enable real-time monitoring of their distribution within the artery. In another study, superparamagnetic iron oxide nanoparticles (SPIONs) were synthesized by Fan's group [213] and mixed with acrylic monomers to form SPION-loaded polymeric microspheres (SPMs). These microspheres exhibited good detectability under MRI and are expected to improve the safety and effectiveness of embolic therapy.

Ultrasound imaging is another convenient and safe method. Wood et al. [214] developed a thermosensitive gel drug delivery system based on perfluorobutane microbubbles (MBs) and poloxamer. This system enhanced multimodal imaging navigation, integrating ultrasound and X-ray/CT imaging to improve tumor targeting, spatial drug delivery, and visualization of the tumor and gel overlap area. Additionally, Yang's team [121] explored the potential of a Pickering emulsion of 2H, 3H-decafluoropentane (HDFP) for long-term ultrasound imaging, evaluating its application as an ultrasound-guided embolic material in TAE (Fig. 11c). Zheng et al. [215] introduced a dual-mode imaging material composed of dimethoxytetraphenylethylene (DMTPE), poly (N-isopropylacrylamide-acrylic acid) (PNA), iodized oil, and fluorocarbon compounds. This material demonstrated stable contrast, good fluidity, and embolic properties (Fig. 11d).

Radionuclide-based imaging systems also show promising potential. Yi et al. [130] prepared microspheres cross-linked with PVA macromonomers, N-acryloyltyrosine, and N, N′-methylenebisacrylamide (MBAm). These microspheres were successfully used for embolization of the tumor-feeding artery under ^18^F-FDG PET/CT scanning, providing non-invasive monitoring of the anti-tumor effect of TACE in renal tumors.

## 4 Challenges and clinical translation prospects of injectable hydrogels in tumor therapy

### 4.1 Current status of clinical translation of embolic hydrogels

Embolic hydrogels have been extensively studied over the past few decades, and several successful products have been entered clinical application and approved by the Food and Drug Administration (FDA) for embolization therapy [216]. The hydrogel-based embolic materials available in the market or undergoing clinical studies are detailed in Table 4. Specifically, N-butyl-2-cyanoacrylate (nBCA), commonly known as "glue", has been approved for embolization therapy as a transparent and free-flowing liquid since the mid-1980s [217]. nBCA is primarily used for vascular malformations and hemostasis due to its excellent embolic and hemostatic properties [218,219]. Additionally, Onyx (a new liquid embolic agent, Micro Therapeutics, Inc., Irvine, CA), an in situ precipitated gel composed of ethylene vinyl alcohol polymer and dimethyl sulfoxide solvent, was approved by the FDA in 2005 for the treatment of cerebral arteriovenous malformations (AVMs) [220,221]. Onyx's excellent injectability allows it to penetrate the tumor bed effectively for embolization [219]. Embolic hydrogels have also been widely used in clinical trials for various tumor types, including pancreatic, liver, rectal, prostate, and head and neck cancers. Despite their broad application, current research mainly focuses on improving tumor imaging quality, reducing chemotherapy and radiotherapy side effects, and enhancing the efficacy of local immunotherapy [216]. Purified porcine gelatin is a commonly used temporary embolic agent in clinical practice. Since 1964, gelatin foam (such as Gelfoam® from Pfizer, New York) has been widely utilized in embolic therapy for hypervascular tumors like HCC and uterine fibroids. Gelatin foam helps inhibit tumor growth and reduces arterial rupture and bleeding by blocking the blood supply to tumors [106,222,223]. However, gelatin's uncontrolled degradation can lead to the complete blockage of blood vessels initially, with potential recanalization of tumor blood vessels as gelatin degrades, increasing the risk of tumor recurrence [224]. To address this limitation, researchers developed triacryloyl gelatin microspheres by cross-linking gelatin with triacryloyl groups, resulting in Embosphere microspheres (Merit Medical), which were FDA-approved for embolization therapy in 2000 [225,226]. Embosphere microspheres have uniform size and smooth contours, making them suitable for delivery through microcatheters [227]. Their broad particle size distribution enables them to embolize both large and small blood vessels [228]. Additionally, PVA hydrogel microspheres have good biocompatibility and compressibility, allowing for the selection of different-sized microspheres for embolization based on clinical needs [229,230]. Bead Block® (Biocompatibles Ltd, UK) and LC Bead microspheres (Biocompatibles Ltd, UK) are FDA-approved for the embolization of highly vascular tumors and AVMs [6,231].

**Table 4:** Embolic hydrogel microspheres approved or undergoing clinical trials.

Materials	Drug delivery	Diseases	Clinical approval
TANDEM® cryospheres	DOX	HCC	Stage III
PVA hydrogel beads	DOX	HCC	Stage IV
Life Pearls microspheres	Irinotecan	HCC/CRC	Stage II
Embozene microspheres	DOX	HCC	2015
CalliSpheres microspheres	DOX/EPI/GEM	HCC	2018
EvenSpheres microspheres	DOX	HCC	2021
LC Bead LUMI™	–	Hypervascular tumors and AVMs	2016
DC Bead LUMI™	–	Hypervascular tumors and AVMs	2017
Gelatin sponge microparticles	–	Hypervascular tumors and arterial hemorrhagic lesions	1969

Drug-eluting beads (DEBs), made from sulfonate-modified PVA hydrogels, utilize negatively charged hydrogel microspheres that can bind to positively charged chemotherapy drugs (such as DOX) through ion exchange, facilitating drug loading and release [232]. DEBs containing sulfonic acid groups (such as DC Bead®) or sodium acrylate groups (such as HepaSphere™ microspheres) are currently in clinical use [233]. These beads not only block tumor blood vessels to cause ischemic necrosis but also promote tumor cell death through drug release, showing excellent tumor control rates in clinical trials [[234], [235], [236]]. HepaSphere™ microspheres, a new type of drug-controlled release embolic material, can load positively charged anti-tumor drugs through surface charge modification. These microspheres have been widely used in tumor embolization therapy and have demonstrated promising results [[237], [238], [239]]. Radiation therapy is limited in anti-tumor treatment due to its potential damage to normal tissues. Selective delivery of radioembolic microspheres to tumor-feeding arteries significantly enhances anti-tumor efficacy while ensuring safety [240]. For example, SirSpheres® consists of biodegradable resin-based microspheres with an average diameter of approximately 30 μm, with a^90^Y radioisotope attached to the surface for radioembolization therapy [166,241]. This product was FDA-approved in 2002 for treating advanced HCC and colorectal cancer liver metastases [242,243].

### 4.2 Hydrogel-based embolization agents: comparison and clinical translation challenges

#### 4.2.1 Comparison between hydrogel embolic agents and traditional embolic agents

In considering the clinical translation of embolic agents, it is essential to compare hydrogel embolic agents with traditional liquid embolic agents like nBCA and Onyx. While traditional liquid embolic agents are widely used in clinical treatments and are effective in achieving embolization, they have inherent limitations in terms of injectability, coagulation time, and long-term stability.

Traditional liquid embolic agents like nBCA and Onyx are characterized by their fast coagulation times. NBCA begins to coagulate almost instantly (within ∼0.1 s), while Onyx coagulates after about 2 min upon contact with blood [217,221]. Although these rapid coagulation properties facilitate immediate vascular occlusion, they also present operational challenges, such as the potential for catheter blockage, improper embolization, or incomplete occlusion due to the difficulty in controlling the timing and location of solidification [6]. Additionally, Onyx contains DMSO, which, when injected too rapidly, may cause vascular damage [244]. These challenges underline the need for embolic agents with controlled gelation kinetics that allow more precise manipulation during the procedure.

The injection velocity of embolic materials plays a pivotal role in their clinical performance, particularly in tumors with fine peripheral vessels. Hydrogel embolic agents have a distinct advantage in this regard due to their shear-thinning behavior, which enables precise control over injection velocity and distal diffusion. Faster injection can promote deeper penetration into small-caliber vessels, but excessively high velocity may increase the risk of non-target embolization or uncontrolled distal migration. Conversely, slower injection may cause premature gelation or incomplete embolization. Hydrogel embolic agents offer the flexibility to adjust injection velocity more accurately, which is critical for achieving complete vascular occlusion in complex tumor vasculature [6,18,107].

Hydrogel embolic agents offer superior control over gelation time, a crucial parameter for optimizing drug release and embolization precision. For instance, the gelation time of pNIPAAm hydrogel at physiological temperature ranges from 5 to 10 min [45], while PLGA-PEG-PLGA block copolymer hydrogels gel within 10 min at body temperature [54]. In contrast, liquid embolic agents like nBCA and Onyx exhibit rapid gelation that can cause operational difficulties [244,245]. The ability to finely tune the gelation time of hydrogels ensures better control over the embolization process, reducing the risks of premature clotting or incomplete vascular occlusion.

Hydrogel embolic agents are generally characterized by lower injection forces compared to traditional liquid embolic agents. For instance, CMC-MA hydrogels require 16.3 N under a 26-gauge needle, while STH hydrogels require 8–34 N, which is significantly lower than the ∼40 N required for Onyx [71,114]. According to literature, liquid embolic agents with an injection force of less than 50 N can be easily delivered through a catheter, while forces between 50 and 100 N still allow moderate resistance [246,247]. For forces exceeding 100 N, injection becomes difficult and unstable. Hydrogel embolic agents, therefore, offer improved maneuverability and clinical safety during transcatheter delivery, enhancing their potential for routine clinical use.

Hydrogel embolic agents also offer significant advantages in terms of imaging visibility, which is critical for real-time monitoring during embolization. The high hydration and three-dimensional network structure of hydrogels enable real-time visualization using techniques such as MRI, X-ray, or ultrasound imaging. For example, hydrogels such as Bi_2_S_3_@SH microspheres offer T_2_-weighted MRI imaging capabilities, providing better image contrast and real-time monitoring compared to traditional liquid embolic agents like Onyx [210,212]. This enhanced imaging ability improves treatment precision, allowing dynamic adjustments during the procedure to optimize therapeutic outcomes.

Building on the above considerations regarding injection dynamics and imaging-guided control, recent advances in catheter-based delivery technologies have further expanded the operability and clinical applicability of hydrogel embolic systems. In parallel with the rapid development of hydrogel embolic materials, their delivery technologies and device designs have also evolved to meet the clinical demands for controllable gelation, stable delivery, and precise embolization. For instance, dual-syringe systems and coaxial double-lumen microcatheters have been increasingly applied in the administration of calcium alginate hydrogels and the FDA-cleared Embrace™ Hydrogel Embolic System [248,249]. These devices enable the separate delivery of precursor solutions and crosslinking agents, ensuring in situ gelation while preventing premature solidification within the catheter. For thermosensitive PNIPAM-based hydrogels, simultaneous cold saline perfusion during injection has been used to transiently suppress gelation, thereby prolonging the injectability window and allowing deeper distal penetration before temperature-triggered solidification [18]. In addition, photo-crosslinkable hydrogels have stimulated the development of optical-fiber-integrated microcatheters, which allow real-time irradiation and on-demand crosslinking at targeted vascular sites [82,182]. This integration of material innovation with advanced delivery platforms provides more refined control over injectability, gelation timing, and embolization precision, and is expected to further enhance the clinical applicability and safety of hydrogel embolic agents.

Recently, several clinically approved hydrogel embolic systems have been introduced, including FDA-approved products and the world's first thermosensitive hydrogel embolic agent independently developed in China. From the FDA-approved portfolio, Obsidio™ Conformable Embolic (developed by Boston Scientific) and Embrace™ Hydrogel Embolic System (by MicroVention) have been designed to address limitations of traditional embolic agents. The Obsidio™ Conformable Embolic System utilizes a biodegradable gelatin-based hydrogel combined with laponite and tantalum for enhanced radiopacity. This system undergoes controlled gelation in the physiological environment, allowing for precise embolization with tailored gelation times that optimize therapeutic outcomes [250]. The formulation offers enhanced injectability, minimizing catheter friction during delivery, and providing sustained occlusion in highly vascularized tumors. Obsidio™ has demonstrated effectiveness in controlled drug release and long-term stability [251,252]. However, it is important to acknowledge a recent FDA Class I recall in 2024 for Obsidio™ Conformable Embolic, triggered by an increased risk of bowel ischemia when used for treating lower gastrointestinal bleeding. This recall resulted from 11 adverse events, including 7 patient injuries and 2 deaths, as reported by the FDA [253,254]. This safety update is incorporated to present a comprehensive clinical safety profile of the agent, aligning with the practical relevance of embolic materials in clinical practice. The Embrace™ Hydrogel Embolic System (HES; Instylla, Bedford, MA, USA) is a biocompatible, hydrophilic polymeric hydrogel designed for controlled, safe, and targeted embolization therapy [248]. This system is based on polyethylene glycol (PEG) that rapidly solidify into a soft hydrogel upon mixing within the bloodstream.Embrace™ offers precise gelation control, enabling accurate embolization even in complex tumor vasculature. Clinical investigations have confirmed the safety and efficacy of Embrace™ HES, making it a promising option for tumors requiring precise and controlled embolization therapy [255]. Notably, TempSLE™, a thermosensitive hydrogel based on poly(N-isopropylacrylamide) (PNIPAM) copolymers, represents a milestone in the independent innovation of embolic materials in China. It is the first thermosensitive hydrogel embolic agent independently developed in China and was approved by the National Medical Products Administration (NMPA) in 2020 [44]. Leveraging the temperature-responsive sol–gel transition of PNIPAM copolymers, TempSLE™ exhibits a finely tuned gelation window, achieving a sol–gel transformation within 10–15 s after exiting the catheter. This property provides an optimal balance between injectability and rapid post-delivery occlusion, effectively overcoming the procedural limitations of conventional liquid embolic agents [256]. When drug-loaded TempSLE™ is administered *in vivo*, its thermoresponsive behavior enables site-specific drug release and sustained drug availability, maintaining a stable plasma concentration. This design minimizes burst release, reduces systemic side effects, and enhances bioavailability. Clinical evidence has demonstrated its superiority in TACE for HCC, showing a reduced catheter clogging rate and an improved disease control rate (≥90 %) [257]. These features make TempSLE™ a representative advancement in the development of hydrogel-based embolic agents.

Beyond the currently approved systems, several next-generation hydrogel embolic agents are undergoing clinical evaluation under the supervision of the FDA and NMPA, reflecting the rapid clinical translation of this technology. Among these, alginate-based injectable hydrogels have attracted extensive attention due to their excellent biocompatibility, favorable injectability, and well-established safety record. Alginate has been classified by the FDA as “Generally Recognized as Safe” for biomedical applications [14]. A representative example is EmboGel™, a radiopaque, alginate-based embolic biomaterial that has demonstrated favorable embolization performance in preclinical and early clinical investigations [258]. In parallel, Obsidio™ Conformable Embolic remains under post-market surveillance and additional clinical evaluations aimed at refining its indications and reducing ischemic complications associated with non-tumor embolization procedures. In China, several advanced hydrogel embolic platforms are progressing toward clinical translation. These include PEG-PCL-PEG triblock copolymer hydrogels and chitosan-based thermo-gelling embolics, both of which have shown promising results in preclinical studies targeting hepatocellular carcinoma and uterine fibroid embolization [259]. Another noteworthy candidate is the alginate hydrogel embolic system, designed to combine intrinsic radiopacity with controlled biodegradability, thereby enhancing both imaging visibility and long-term safety [260]. Collectively, these ongoing developments underscore a global effort to engineer hydrogel-based embolic systems with improved biocompatibility, real-time imaging capability, and multifunctional therapeutic potential, laying a solid foundation for the next generation of clinically translatable embolization therapies.

In summary, hydrogel embolic agents offer clear advantages over traditional liquid embolic agents in terms of injectability, gelation time control, and imaging capability, with approved products (including China's innovative TempSLE™) and ongoing clinical trials further solidifying their potential for tumor embolization therapies. Nevertheless, clinical translation requires continued evaluation of mechanical strength (to withstand vascular pressure fluctuations), long-term biocompatibility (to avoid chronic inflammation), and scalable manufacturing (to ensure batch consistency)-factors that will determine their long-term success in routine clinical practice.

#### 4.2.2 Challenges in the clinical translation of embolic hydrogels

**Fig. 12:**
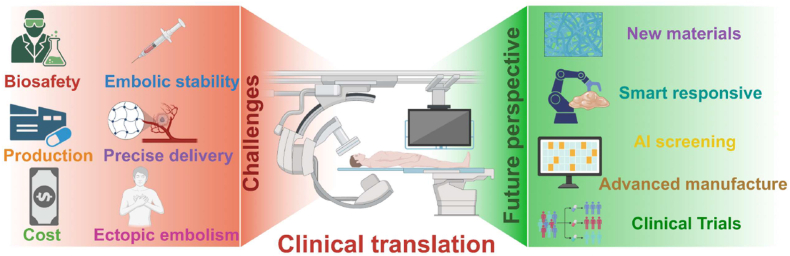
Challenges and clinical translation prospects of embolic hydrogels in tumor therapy. Created by BioRender.com.

While injectable hydrogels have demonstrated considerable promise in biomedical applications, they are still largely in the preclinical phase, with major obstacles remaining in areas such as biosafety, large-scale manufacturing, and regulatory approval processes (Fig. 12) [68,156].

One of the foremost challenges in clinical translation is biosafety. Many hydrogel components, such as quaternized chitosan, tannic acid, and novel cross-linkers, are yet to receive FDA approval, posing risks such as inflammatory reactions, cytotoxicity, or ectopic embolization upon implantation [9,18]. The HydroCoil Embolic System (HES), a hydrogel-coated platinum coil approved for treating intracranial aneurysms, has faced issues with long-term stability and mechanical properties of the hydrogel coating [261]. This example underscores the complexity of maintaining the stability and functionality of hydrogel coatings in clinical use. In contrast, FDA-approved embolic materials such as nBCA have been widely used in embolization therapy and demonstrate excellent embolic properties. However, they are hindered by poor mechanical stability, which complicates the embolization process and increases the risk of recanalization [217]. Additionally, Onyx is effective in embolization but faces similar concerns regarding the long-term stability of treated vessels [219]. Furthermore, a PEG-based liquid embolic hydrogel recently completed first-in-human clinical trials in patients with hypervascular tumors, providing valuable insights into translational opportunities and barriers [255]. These cases emphasize that stability, biodegradability, and the risk of unintended embolization or tissue damage must be carefully considered when designing embolic hydrogels.

Large-scale production and industrialization present another challenge. Embolic hydrogels need to be produced with high uniformity to ensure consistent performance in clinical settings. Variations in raw materials, such as chitosan or hyaluronic acid, can lead to inconsistent hydrogel properties, affecting embolization efficiency [158]. Furthermore, sterilization methods must be carefully optimized, as traditional high-pressure sterilization (e.g., autoclaving) can disrupt the three-dimensional network of hydrogels due to their high-water content and thermal sensitivity, resulting in altered viscosity, premature gelation, or loss of mechanical integrity [224]. To address these limitations, clinically relevant hydrogel embolic agents typically employ γ-irradiation, ethylene oxide (EtO) sterilization, sterile filtration (for precursor solutions), or fully aseptic manufacturing processes [262,263]. γ-irradiation is widely used for pre-crosslinked or lyophilized hydrogels due to its strong penetrability and minimal impact on polymer backbone stability, while EtO sterilization is applicable to temperature-sensitive systems but requires careful removal of chemical residues to avoid vascular toxicity. For in situ-forming hydrogels, sterile filtration combined with aseptic filling is preferred to maintain precursor purity without altering rheological or gelation characteristics [9]. These sterilization strategies are increasingly incorporated into Good Manufacturing Practice (GMP) workflows to support clinical translation.

In addition to sterilization, storage stability and shelf-life control are critical considerations for embolic hydrogels. Depending on polymer composition and gelation mechanisms, hydrogels may be stored as (i) pre-crosslinked hydrogels pre-loaded into syringes, (ii) lyophilized powders reconstituted before use, or (iii) dual-chamber systems containing separated precursor solutions to prevent premature gelation. Hydrogels formulated in a hydrated, ready-to-use format must maintain stable viscosity and gelation behavior throughout storage [264]. For example, Obsidio™ is supplied as a pre-filled syringe with a validated shelf life of 1 year, demonstrating the feasibility of pre-loaded hydrogel systems under controlled storage conditions (typically room temperature or 2–8 °C, depending on polymer stability) [250,251]. Dual-syringe or dual-lumen catheters further enhance storage stability for ionically or chemically crosslinked hydrogels by separating reactive components until administration. These storage strategies ensure that embolic hydrogels maintain batch-to-batch uniformity, sterility, and functional performance during clinical use. Triacryloyl gelatin microspheres, such as Embosphere microspheres, have been developed to address sterilization and storage challenges and have been FDA-approved for embolization. However, the biodegradation of gelatin remains a concern that must be resolved to improve long-term effectiveness [225,226]. Overall, the establishment of robust sterilization protocols, storage formats, and GMP-compliant manufacturing processes is essential for ensuring the clinical reliability and translational viability of hydrogel embolic agents.

A significant challenge in the clinical translation of embolic hydrogels lies in regulatory approval. Given their direct implantation into blood vessels, these hydrogels must meet stringent FDA standards for homogeneity, stability, and biocompatibility. The approval process requires extensive clinical trials to demonstrate both safety and efficacy, which is costly and time-consuming. Products like SirSpheres®, a biodegradable resin-based microsphere for radioembolization, have been FDA-approved for advanced HCC and colorectal cancer liver metastases after comprehensive studies [242,243]. These regulatory requirements are essential for patient safety and therapeutic efficacy but also present a significant barrier for new hydrogel systems seeking approval.

Another critical challenge is the assessment and prevention of ectopic embolization. Off-target embolization may occur when low-viscosity precursors or mechanically unstable hydrogels migrate into non-target organs. Major risk factors include insufficient gelation rate, inadequate mechanical strength, suboptimal injection velocity, and patient-specific hemodynamic variations at the injection site [17,265]. Comprehensive evaluation strategies involve complementary in vitro, *in vivo*, and imaging-based methodologies. In vitro perfusion models constructed with anatomically representative vascular phantoms enable simulation of physiologic shear forces and bifurcation flow patterns, allowing quantification of distal migration thresholds. *In vivo* renal or hepatic artery embolization models in rats or rabbits further provide evidence of hydrogel retention, off-target distribution, and tissue response [185]. Real-time imaging modalities-including fluoroscopy, MRI, and CT-are indispensable for monitoring hydrogel localization and ensuring early detection of unintended migration [9,266]. Incorporating radiopaque fillers or MRI-active components significantly enhances visualization, enabling dynamic adjustment of injection parameters to improve procedural safety.

For biodegradable hydrogel embolic agents, understanding metabolic pathways and degradation kinetics is essential to balance embolization durability with biosafety. Hydrogel degradation *in vivo* is governed by polymer composition, crosslinking mechanisms, enzymatic susceptibility, and local microenvironmental factors such as pH or ROS [49,89,119,266]. Natural-polymer hydrogels (e.g., gelatin, hyaluronic acid, chitosan) typically undergo enzyme-mediated cleavage by matrix metalloproteinases, hyaluronidases, or lysozymes, generating oligosaccharides or peptides that are cleared via renal or hepatic routes. Synthetic biodegradable systems-such as PLGA-PEG-PLGA or poly(ester)-based hydrogels-primarily degrade through ester hydrolysis, producing lactic and glycolic acids that enter physiological metabolic pathways including the tricarboxylic acid cycle [54,55].

Finally, the industrialization of embolic hydrogels will require overcoming substantial barriers, including GMP production, scalable fabrication technologies, and navigating regulatory approval processes. A key area of focus will be the development of novel materials that can enhance the stability, uniformity, and performance of hydrogels. For example, hydrogels with stimuli-responsive properties, such as those that respond to pH, temperature, or ROS in the TME, show great promise for enhancing tumor-targeted embolization and minimizing off-target effects [68,158]. In addition, the incorporation of artificial intelligence (AI) and machine learning in the development of embolic hydrogels can provide valuable insights into optimizing their synthesis and improving their precision. AI-driven high-throughput screening platforms could be employed to design and evaluate hydrogel formulations, simulating embolic behaviors and mechanical changes during embolization, thereby streamlining the design and manufacturing processes [185].

In conclusion, while embolic hydrogels hold great promise for tumor embolization, their clinical translation is impeded mainly by challenges in biosafety, large-scale production, and regulatory approval. Overcoming these challenges, alongside the development of hydrogel formulations with enhanced stability and functionality, will be crucial for advancing their clinical application in tumor therapy.

### 4.3 Application prospects of embolic hydrogels

With ongoing advancements in embolic hydrogel preparation technology and industrialization, hydrogel embolization platforms are increasingly being integrated into the biomedical field. One key approach to expanding their application is the development of novel materials for hydrogel preparation. These materials are expected to address challenges related to stability and uniformity during preparation, thereby enhancing the embolic performance of hydrogels [39,40]. Additionally, the development of intelligently responsive hydrogels has emerged as an important research direction. Hydrogels that respond to factors such as pH, ROS, temperature, and mechanical strength within the TME can enable precise, targeted embolization, improving treatment specificity for target organs and tissues.

As high-throughput screening platforms and AI technologies continue to evolve, integrating AI into the hydrogel development process presents exciting opportunities. For example, recent studies such as “AI energized hydrogel design, optimization and application in biomedicine” demonstrate how machine-learning models trained on hydrogel composition-structure-property databases can predict gelation kinetics, swelling behaviour and mechanical modulus, thereby accelerating the discovery of candidate formulations [267]. Another work, “Exploring the potential of artificial intelligence for hydrogel development-A short review”, outlines how neural networks and support vector machines are now being applied to relate polymer/cross-linker chemistries with functional outputs such as degradation rate and drug-release behaviour [268]. A further concrete example is the study applies Bayesian-optimization algorithms to tune dual-network hydrogels for desired mechanical and sensing properties, illustrating how AI-driven optimization can compress the experimental cycle [269]. In the context of embolic hydrogels, these AI-enabled workflows may be adapted to simulate injectability, distal diffusion and gel-stability under flow conditions, enabling formulation-delivery design iterations that match interventional catheter constraints and vascular shear environments. As multidisciplinary collaboration between materials scientists, oncologists, and interventional physicians becomes increasingly essential, the integration of clinically-relevant parameters (e.g., injection force, gelation window, vascular shear) into AI training datasets will help tailor new embolic hydrogels explicitly for tumor-embolization applications. By designing new embolic hydrogels tailored to clinical needs and leveraging existing materials, these collaborative efforts will help address the obstacles faced by embolic hydrogels, advancing their clinical application.

## 5 Conclusions

In summary, embolic hydrogels represent a promising class of biomaterials for cancer treatment, offering a unique combination of precision in drug delivery and vascular embolization. These hydrogels, through various gelation pathways, can meet specific embolic requirements, enhancing the effectiveness of embolization therapies. Their superior drug delivery capabilities and microenvironment-responsive properties enable controlled and sustained release within tumors, optimizing therapeutic outcomes. The integration of injectable hydrogels with minimally invasive interventional techniques further elevates their potential in tumor therapy, improving drug delivery precision. Notably, recent advancements have enabled real-time imaging, enhanced drug release, stable vascular embolization, and the integration of multimodal therapies, providing a new paradigm for anti-tumor treatments. Despite these promising advancements, embolic hydrogels still face challenges such as mechanical stability, controlled drug release, biosafety, large-scale production, and clinical translation. Future research should focus on addressing these challenges and advancing the personalized design of intelligent hydrogels, as well as exploring the synergistic mechanisms of multimodal therapeutic strategies, to fully realize their potential in precision medicine and personalized cancer treatment.

## CRediT authorship contribution statement

**Yisheng Peng:** Writing – original draft, Visualization, Conceptualization. **Xiuyi Wu:** Writing – original draft, Visualization. **Hui Liu:** Writing – original draft, Resources. **Fengyi Yang:** Writing – original draft, Resources. **Xu Cheng:** Writing – original draft, Visualization. **Mengmeng Miao:** Visualization. **Shangqing Chen:** Resources. **Kaifei Yan:** Resources. **Hui Zheng:** Writing – review & editing. **Hongwei Cheng:** Writing – review & editing, Writing – original draft, Visualization, Supervision, Funding acquisition, Conceptualization. **Gang Liu:** Writing – review & editing, Supervision, Funding acquisition, Conceptualization.

## Ethics approval and consent to participate

This is no ethics approval and consent to participant involved in this article.

## Declaration of competing interest

The authors declare that they have no known competing financial interests or personal relationships that could have appeared to influence the work reported in this paper.

## Abbreviations:

hepatocellular carcinoma (HCC), digital subtraction angiography (DSA), Computed Tomography (CT), single-photon emission computed tomography (SPECT), Magnetic Resonance Imaging (MRI), tumor microenvironment (TME), Doxorubicin (DOX), transcatheter arterial embolization (TAE), transarterial chemoembolization (TACE), transarterial radioembolization (TARE), renal artery embolization (RAE), Uterine artery embolization (UAE), photothermal therapy (PTT), Magnetic hyperthermia therapy (MHT), reactive oxygen species (ROS), immunogenic cell death (ICD), immune checkpoint inhibitors (ICIs), Food and Drug Administration (FDA), arteriovenous malformations (AVMs), Good Manufacturing Practice (GMP), artificial intelligence (AI), Poly(N-isopropylacrylamide) (pNIPAAm), Polyethylene Glycol (PEG), Polyvinyl Alcoho (PVA), polydopamine (PDA), N-butyl-2-cyanoacrylate (nBCA), Drug-eluting beads (DEBs), Onyx (a new liquid embolic agent, Micro Therapeutics, Inc., Irvine, CA).
